# Nonpharmacological interventions on glycated haemoglobin in youth with type 1 diabetes: a Bayesian network meta-analysis

**DOI:** 10.1186/s12933-024-02301-3

**Published:** 2024-07-01

**Authors:** Jacinto Muñoz-Pardeza, José Francisco López-Gil, Nidia Huerta-Uribe, Ignacio Hormazábal-Aguayo, Mikel Izquierdo, Antonio García-Hermoso

**Affiliations:** 1 Navarrabiomed, Hospital Universitario de Navarra, Universidad Pública de Navarra (UPNA), IdiSNA, Pamplona, Spain; 2 One Health Research Group, Universidad de Las Américas, Quito, Ecuador; 3 CIBER of Frailty and Healthy Aging (CIBERFES), Instituto de Salud Carlos III, Madrid, Spain

**Keywords:** Adolescence, Childhood, Diet, Exercise, Glucose, Glycosylated haemoglobin, Insulin, Intervention, Lifestyle, Nonpharmacological

## Abstract

The available evidence on the impact of specific non-pharmacological interventions on glycaemic control is currently limited. Consequently, there is a need to determine which interventions could provide the most significant benefits for the metabolic health of young individuals with type 1 diabetes mellitus. The aim of this study was to identify optimal nonpharmacological interventions on glycaemic control, measured by glycated haemoglobin (HbA1c), in children and adolescents with type 1 diabetes. Systematic searches were conducted in PubMed, Web of Science, Scopus, and SPORTDiscus from inception to July 1, 2023. Randomised clinical trials (RCT) investigating nonpharmacological interventions (e.g., physical activity, nutrition, and behavioural therapies) were included. Primary outcome was change in HbA1c levels. Secondary outcome was change in daily insulin dose requirement. Seventy-four RCT with 6,815 participants (49.43% girls) involving 20 interventions were analysed using a network meta-analysis. Most interventions showed greater efficacy than standard care. However, multicomponent exercise, which includes aerobic and strength training (n = 214, standardised mean difference [SMD] =– 0.63, 95% credible interval [95% CrI] – 1.09 to – 0.16) and nutritional supplements (n = 146, SMD =– 0.49, – 0 .92 to – 0.07) demonstrated the greatest HbA1c reductions. These interventions also led to the larger decreases in daily insulin needs (n = 119, SMD =– 0.79, 95% CrI –  1.19 to – 0.34) and (n = 57, SMD =– 0.62, 95% CrI –  1.18 to – 0.12, respectively). The current study underscores non-pharmacological options such as multicomponent exercise and nutritional supplements, showcasing their potential to significantly improve HbA1c in youth with type 1 diabetes. Although additional research to confirm their efficacy is required, these approaches could be considered as potential adjuvant therapeutic options in the management of type 1 diabetes among children and adolescents.

**Supplementary Information:**

The online version contains supplementary material available at 10.1186/s12933-024-02301-3.

## Introduction

Type 1 diabetes is an inflammatory autoimmune disease characterized by progressive damage to pancreatic β-cells, leading to insulin deficiency [1]. Type 1 diabetes can occur at any age [2] and affects approximately 1.2 million children and adolescents annually [3]. Type 1 diabetes increases risks of microvascular and macrovascular complications [4–7], making glycaemic control crucial. Glycated haemoglobin (HbA1c) strongly associates with complications and predicts long-term health [8], with levels below 7% recommended to reduce vascular complications and mortality [9, 10].

The American Diabetes Association (ADA) and International Society for Paediatric and Adolescent Diabetes (ISPAD) recommend multifaceted care for paediatric type 1 diabetes, including nutrition therapy, physical activity and exercise, glucose monitoring, insulin therapy, education, and psychosocial support [7, 11]. However, evidence regarding the efficacy of certain non-pharmacological interventions remains limited and conflicting. Research suggests that performing 60 min of moderate to vigorous PA and engaging in strengthening activities three non-consecutive days per week can reduce HbA1c levels and daily insulin requirements, improving metabolic control [7, 12, 13]. Nonetheless, despite effective strategies focused on carbohydrate counting to decrease HbA1c levels, the evidence regarding optimal calorie distribution, nutrients and supplements intake remains inconclusive [14–17]. Similarly, although educational and psychological interventions aim to increase family involvement and promote self-management skills to improve diabetes management, their metabolic benefits are debated [14, 15].

Currently, there is a need for a comprehensive synthesis of nonpharmacological interventions for youth with type 1 diabetes through network meta-analysis (NMA), as this is currently lacking. By comparing direct and indirect intervention effects on HbA1c, NMA can elucidate the most effective strategies for paediatric care. This study tried to identify optimal nonpharmacological interventions to reduce HbA1c levels as a primary outcome, and daily insulin requirements as a secondary outcome, compared to usual care conditions in children and adolescents with type 1 diabetes.

## Methods

Two researchers (JM-P and NH-U) independently carried out a rigorous systematic exploration to replicate the number of studies initially obtained. Any disparities found in the collection process were reconciled through consensus discussions involving a third researcher (AG-H).

### Protocol and registration

The study was conducted according to the NMA extension of the Preferred Reporting Items for Systematic Reviews and Meta-Analyses (PRISMA-NMA) guidelines [18]. The study protocol was registered in PROSPERO (CRD42023432586).

### Eligibility criteria

The studies were included if they met all of the following criteria: (i) the mean age of the study cohort was required to be 18 years or younger, with a confirmed diagnosis of type 1 diabetes mellitus, regardless of disease duration; (ii) the study assessed a group enrolled in at least one of the following nonpharmacological intervention: physical exercise program, physical activity promotion, dietary interventions, nutritional education, behavioural therapy, or diabetes education; (iii) the experimental group was compared to a control group or another different intervention; (iv) the HbA1c measurement was one of the dependent variables; and (v) the study design was a randomised clinical trial (RCT).

Exclusion criteria were: (i) having comorbidities other than diabetes, (ii) receiving the same type of intervention between groups (e.g., two similar dietary interventions), and (iii) gray literature (e.g., conference proceedings or editorials). No specific criterion regarding the minimum duration of the program was set.

### Information sources and search

Relevant articles were identified by title and abstract in the electronic databases PubMed, Web of Science, Scopus, and SPORTDiscus from inception to July 1, 2023. Search terms related to the disease “Diabetes Mellitus Type 1” were used. No limitation according to the date of publication was done.

Terms related to the dependent variable of interest and its abbreviation, glycated haemoglobin or HbA1c or A1c, were searched. Moreover, terms were included to specify the respective interventions (e.g., “exercise”, “physical activity”, “diet”, “nutritional education”, “behavioural therapy”, “diabetes education”, among others). All search strategies are shown in ESM Table 1. Additionally, a comprehensive literature review was conducted, which encompassed the identification of previous reviews addressing similar topics. Therefore, a rigorous analysis of additional studies was carried out using backward citation searching.

### Study selection

First, two of the reviewers (J-MP and A-GH) inputted the citations gathered from the databases into the EndNote bibliographic management tool, and after removing duplicates, the articles were initially screened based on their titles and abstracts. Subsequently, the full text of the studies that met the criteria was evaluated. Each author generated individual lists of the selected studies at each stage. Once again, any discrepancies were resolved by a third researcher (N-HU) to achieve the final compilation of studies.

Interventions obtained were classified as: "Control (CON)", "Aerobic exercise training (AER)", "Behavioural therapy (BT)", "Carbohydrate counting (CC)", "Diabetic education (DEd)", "Diet (DIET)", "Family therapy (FT)", "Multicomponent exercise training (MC)" (e.g., aerobic and strength training), "Nutritional education (NEd)", "Physical activity promotion (PAP)", "Resistance exercise training (RT)", "Sleep education (SLEd)", "Nutritional supplements (SUP)", "AER + DIET", "BT + FT", "DEd + AER + DIET", "DEd + BT", "DEd + BT + FT", and "NEd + BT", "RT + SUP". The explanation of the independent categories is explained in ESM Table 2. This systematic categorisation facilitated the grouping of studies into 20 distinct categories, enabling a comprehensive approach for conducting NMA [19].

### Data collection process and data items

Two reviewers (J-MP and J-LG) established a database to extract results across four domains. The first encompassed study characteristics including authors, journal, and publication year. The second comprised participant demographics such as sex and age. The third involved intervention details like type, duration, and frequency. The fourth contained HbA1c data as the primary outcome and, when available, daily insulin dose requirements as a secondary outcome.

During data collection, the measurement units used in each study were noted (i.e., %, mmol/mol, U, and U/Kg). To maintain methodological consistency in our analysis and to precisely evaluate the actual effects of the interventions, the extracted data encompassed baseline and immediately post-intervention mean values alongside their corresponding standard deviations (SD). Follow-up measurements were excluded from consideration. For trials presenting results in figures, data was extracted using WebPlotDigitizer software (version 4.6). Means and SDs were estimated if needed [20–22].

### Risk of bias within individual studies

Two reviewers (JM-P and IH-A) used pretested forms to assess the studies risk of bias based on the criteria outlined in the Cochrane Risk of Bias Tool 2.0 (RoB 2.0) [23]. This tool comprises five domains that assess aspects such as randomization (D1), implementation of interventions (D2), loss of data (D3), measurement of variables (D4), and reporting of results (D5). The RCTs were analysed individually and assigned as “high risk of bias” if one of the five domains was at high risk of bias; “moderate risk of bias” if at least one of the five domains had possible indications of bias; and “low risk of bias” if there were no concerns for any of the domains. Any disagreement in quality ratings was discussed. If consensus could not be reached, a third member of the review team (JL-G) was consulted.

### Summary measures

Comprehensive meta-analysis software (v3; Biostat, Inc.; Englewood, New Jersey, United States) was used to synthesise the findings and summarise the numerical outcomes of individual studies. The standardised mean differences (Cohen's *d*) (SMD) and the standard error (SE) were computed to determine the effect of interventions and control group independently for each RCT [24]. Subsequently, they were grouped into their respective categories for analysis within the NMA.

### Network meta-analysis

All statistical analyses were carried out using the R statistical software (Version 4.1.1) from R Core Team in Vienna, Austria, and RStudio (Version 2021.09.2) from Posit in Boston, MA, USA. “*BUGSnet*” (Bayesian inference Using Gibbs Sampling to conduct NETwork meta-analysis) is a feature-rich R package to conduct Bayesian NMA in compliance with the best practice and reporting guidelines available [25]. These Bayesian analyses are carried out utilizing Just Another Gibbs Sampler (JAGS). The outputs generated are extensively customizable and encompass a range of visual aids and data summaries. These include network plots, tables detailing network attributes, league tables coupled with league heat plots, Surface Under Cumulative Ranking (SUCRA) plots, rankograms, forest plots, leverage plots, trace plots, and plots for comparing posterior mean deviances. The Confidence in Network Meta-Analysis (CINeMA) framework was employed to ascertain the final outcomes of the NMA, which are contextualised based on the bias of the comparisons, resulting in the certainty of the evidence [26]. CINeMA is a single web application that communicates to an R back-end server [27].

Initially, a narrative synthesis of the RCTs included in this study was performed, compiling the findings in an ad hoc table that delineates both direct and indirect comparisons. Subsequently, to enable the software to analyse the results, the CINeMA guidelines were adhered to by constructing a table in a long and continuous format [27].

Prior to performing the NMA, a meticulous evaluation of three fundamental assumptions was conducted as outlined by previous literature [28]: (a) to avoid potential bias in the comparative analyses, thereby prevention the appearance of heterogeneity and inconsistency, a comprehensive assessment of the likeness and comparability of the trials encompassed by the NMA was performed. The evaluation of similarity was rooted in the comparison of the baseline distribution of variables acknowledged as effect modifiers (e.g., sex, age) among samples within each intervention category; (b) to ensure the homogeneity of conditions associated with the respective studies, a comprehensive verification of the absence of heterogeneity within the outcomes of pairwise comparisons was undertaken. The magnitude and clinical significance of heterogeneity were quantified through the application of the *τ*^2^ statistic, classifying its impact as having either a low degree of clinical relevance (< 0.04), moderate (0.04–0.14), or substantial (0.14–0.40) implications [29]; and (c) a meticulous scrutiny of consistency and transitivity was executed to ensure the absence of significant disparities between direct and indirect evidence. The validity of the similarity assumption or incoherence was ascertained via the implementation of the side or node-splitting methodology [19, 30].

To facilitate the analysis of the results, the following procedures were carried out: robustness was evaluated by employing a network geometry to visually represent the available evidence regarding HbA1c. In this figure, the diameter of the nodes is proportional to the number of participants involved in trials receiving the same type of intervention, and the thickness of the continuous line connecting the nodes is proportional to the number of trials directly comparing a pair of interventions [31].

The comparative assessment of the impact of interventions on HbA1c was executed via a Bayesian NMA, comparing the interactions between interventions and control groups [32]. The relative treatment effect that represents a clinically important difference was defined [26]. In this instance, relative SMD estimates below – 0.8 were regarded as clinically significant [24]; and the range between ± 0.8 is the “range of equivalence”, which corresponds to clinically unimportant differences between interventions. In addition, HbA1c data were reconverted to the same unit of measurement before re-analysis to understand the clinical impact on % HbA1c using mean differences (MD) [33].

To assess inconsistency, we employed the *I*^2^ statistic, which spans the range of 0–100%. Based on the *I*^2^ values, the classification of inconsistency ranged from not important (0–30%), moderate (30–60%), and substantial (60–75%) to considerable (75–100%) [34]. Corresponding *p*-values were also considered.

To identify the most effective intervention, rankograms were generated, which displays the likelihood of each treatment being the optimal choice. Furthermore, we derived estimates for the SUCRA metric for each intervention [31]. The SUCRA allocates a score between 0 and 1 to each intervention within the rankogram, facilitating their ranking. A SUCRA value close to 0 indicates the least favourable intervention, while a value near 1 signifies the most effective intervention. The SUCRA metric synthesises the information regarding the effectiveness of each intervention into a singular value, thereby simplifying the outcomes of NMA into a concise representation. The significance of SUCRA is given when a consistent disparity in preference exists between consecutive ranks throughout the entire rating scale. The SUCRA scores were reported as percentages to improve its interpretation.

### Risk of bias across studies, comparisons and certainty of evidence

The level of confidence in the evidence within coherent networks was evaluated using the Grading of Recommendation, Assessment, Development, and Evaluation (GRADE) methodology. Four degrees of confidence were appraised, ranging from very low to high (reflecting either a strong possibility of substantial variations between the actual and estimated impact or a significant level of assurance in the similarity between the actual and estimated impact, respectively). The GRADE judgments were formulated considering the values yielded by each of the six domains of CINeMA, an online tool was designed by the Cochrane Group, which also aids in evaluating the quality and reliability of evidence generated from the simultaneous comparison of multiple interventions [26]. In accordance with the relevant protocol, each comparison was assessed for potential bias, including within-study bias, reporting bias, directionality, imprecision, heterogeneity and inconsistency. Confidence levels for each domain were initially high, but were subsequently adjusted for the specific methodological issues described below:

#### Within-study bias

Once the RoB 2.0 tool was integrated into the CINeMA framework, the bar chart provided by the system was analysed. Subsequently, the confidence level of each comparison was reduced using the weighted average risk of bias method. To do this, scores of – 1, 0 and 1 were assigned to low, moderate and high risk of bias, respectively [26]. After multiplying each percentage in the bar chart by the corresponding factor and summing the three components, the result of the bias to be reported was obtained.

#### Reporting bias

The Risk of Bias due to Missing Evidence in Network meta-analysis (ROB-MEN) was also used to simplify the assessment of the reporting bias domain associated with CINeMA [35]. This tool incorporates qualitative or quantitative approaches such as funnel plots, tests for small study effects or selection models into an overall assessment for comparative studies such as RCTs.

In addition, the recommendations of the Cochrane Handbook for Systematic Reviews of Interventions [36], Bayesian funnel plots were run to assess publication bias through asymmetry between all nonpharmacological therapies and controls. Independently, the same dynamics were followed for those comparisons with more than 10 studies (behavioural therapy vs control and diabetes education vs control). As these tests are not recommended for comparisons with less than 10 studies, it was decided to indicate “some concerns”.

#### Indirectness

In order to assess whether the available evidence is applicable to the clinical question [37], the weighted average method was selected taking into account the bar chart reported by the framework [27].

#### Imprecision

In the NMA, indirect evidence is added to direct evidence for gaining precision purposes [38]. A SMD = 0.8 was set as indicative of a clinically important effect and then imprecision was assessed using 95% credible intervals (CrI) estimated with the maximum likelihood method. “No concerns” were established if the 95% CrI was entirely on the effect side or entirely within the range of equivalence. The confidence level decreased to “some concern” when 95% CrI covered both clinically important and null effects. Conversely, comparisons were marked as of “major concern” if the 95% CrI limits extended beyond of the opposite equivalence range.

#### Heterogeneity

Considering the clinically significant effect threshold (SMD = 0.8), CINeMA assesses heterogeneity by examining whether the prediction and 95% CrI yield discordant conclusions regarding clinical relevance. Comparisons are considered of “some concern” if the prediction interval enters or leaves the zone of clinical significance once, and of “major concern” if it crosses this region twice.

#### Incoherence

We assessed the ability of the NMA to produce similar estimates when using direct comparisons versus indirect estimates. In addition, we examined the transitivity assumption, which ensures that the only systematic difference between two groups is the treatment and no other confounding factors. Considering the inconsistency factor and using the SIDE method [39], which separates direct and indirect estimates to check for significant differences in effect estimates, we identified “some concern” when direct and indirect 95% CrI reported discrepancies in effect despite to follow the same direction. In contrast, we reported “major concern” when 95% CrI indicated discrepancies due to changes in the direction of effect.

#### Confidence rating

The levels reported by CINeMA in each domain were translated to the levels commonly reported by GRADE as follows: high confidence, indicating that the true effect closely aligns with the estimated effect; moderate confidence, suggesting that the true effect is likely close to the estimate but could differ substantially; low confidence, indicating that the true effect may differ substantially from the estimate; and very low confidence, indicating that the true effect is likely substantially different from the estimate. In the final report, the interrelation between domains was considered, avoiding isolated judgments (e.g., substantial "heterogeneity" may impact increased "imprecision" in the relative treatment effects) [40].

### Additional analyses

We conducted a further NMA using the same methodology to assess how interventions directly and indirectly affected participants daily insulin dose requirements.

In addition, when there were 10 or more studies for the same type of intervention [41], meta-regression analyses were conducted to determine the moderating effect of mean age, number of girls, duration of diabetes since diagnosis, and duration of interventions on the estimates obtained. Moreover, to determine the moderating effect of these same factors in the interventions at the global level, moderation analyses including all interventions (regardless of type) versus control were carried out (following the recommendation of one of the reviewers). All these analyses were performed with the function “*bmr*” of the “*bayesmeta*” package [42].

On the other hand, applying the same criteria as for the meta-regression analyses (i.e., for interventions with 10 or more studies and for interventions at global level), publication bias was assessed through funnel plots (using the function “*funnel*” of the “*bayesmeta*” package).

## Results

### Study selection

**Fig. 1:**
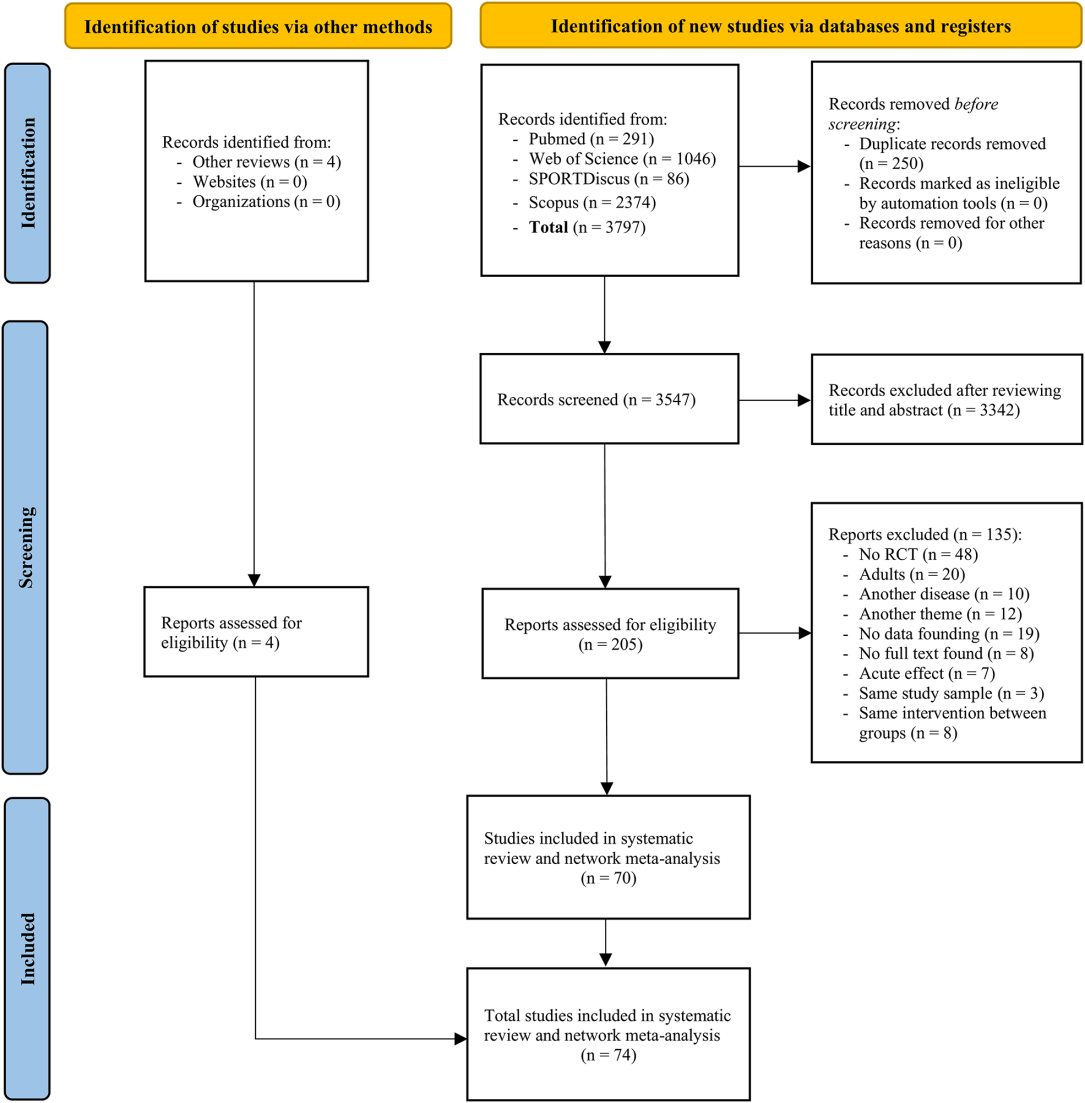
Preferred reporting items for systematic reviews and network meta-analyses (PRISMA-NMA) flow diagram

The initial database search identified 3797 records. After removing duplicates and screening titles/abstracts, 205 articles underwent eligibility assessment. ESM Table 3 details excluded studies and reasons. Ultimately, 70 articles met inclusion criteria and were analysed in the primary NMA on HbA1c, alongside 4 articles from systematic reviews, totalling 74 included studies (Fig. 1).

### Study characteristics

This NMA comprised 6815 children and adolescents with type 1 diabetes (mean age 12.66 ± 1.99 years; 49.43% girls). RCTs were published 1984–2022 across 23 countries on 5 continents. The study characteristics are presented in ESM Methods 1 and ESM Table 4.

### Risk of bias within studies

The RoB 2.0 tool rated 61% of studies as some concerns, 31% as high risk, and 8% as low risk for overall bias (ESM Fig. 1).

### Network meta-analysis results

**Fig. 2:**
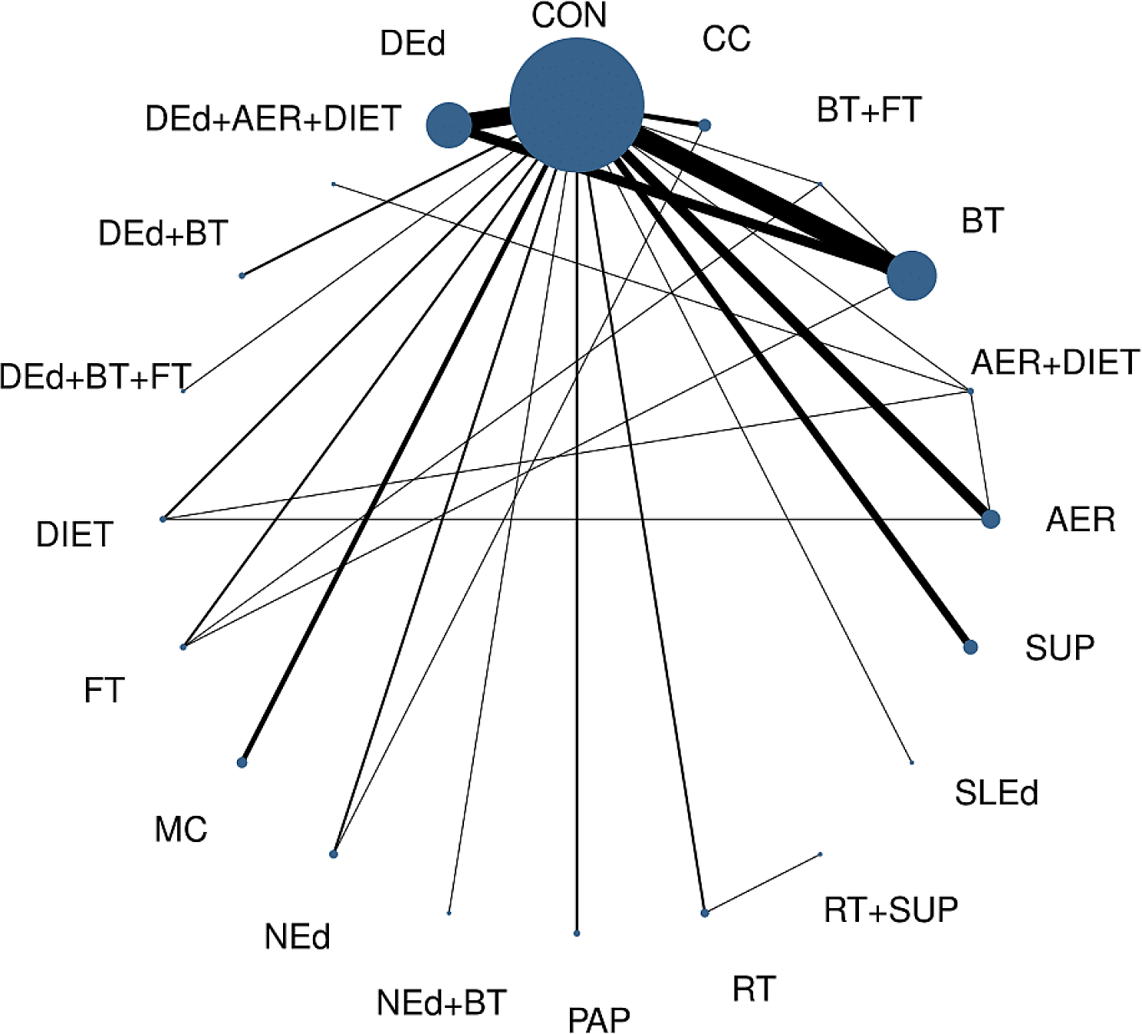
Network plot of available comparisons between different nonpharmacological interventions on glycated haemoglobin (HbA1c) in children and adolescents with type 1 diabetes mellitus. The size of the nodes is directly proportional to the number of participants randomly assigned to each intervention. The width of the connecting lines corresponds to the number of studies comparing the respective interventions. *AER* Aerobic, *BT* Behavioural therapy, *CC* Carbohydrate eounting, *CON* Control, *DEd* Diabetes education, *FT* Family therapy, *MC* Multicomponent, *NEd* Nutritional education, *PA* Physical activity, *RT* Resistance training, *SLEd* Sleep education, *SUP* Nutritional supplements

Figure 2 displays the relative amount of evidence for the 20 interventions compared for HbA1c. ESM Fig. 2 shows the 12 interventions compared for daily insulin dose requirements. In these figures, the nodes represent the sample size for each category, while the lines represent the number of studies comparing the interventions of the respective nodes.

The primary NMA comprised 190 pairwise comparisons (27 mixed, 163 indirect). The secondary NMA involved 66 comparisons (14 mixed, 52 indirect). Table 1 and ESM Table 5 present the detailed results.

**Table 1:**
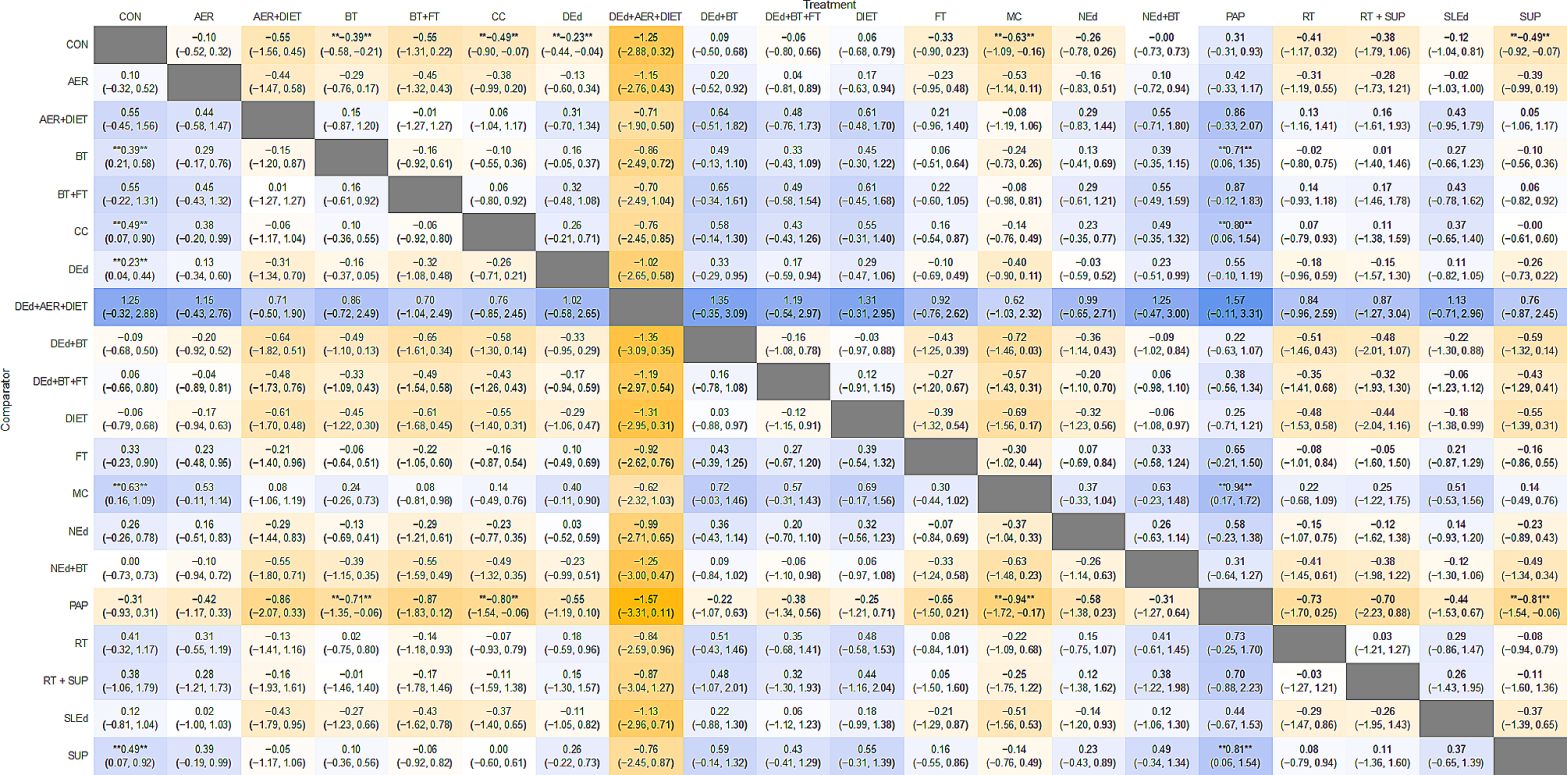
Network meta-analyses of the nonpharmacological interventions on glycated haemoglobin (HbA1c) in children and adolescents with type 1 diabetes mellitus

Values correspond to standardised mean difference and 95% credible interval between column and row. The table is read from top to bottom and from left to right. Darker colours represent a larger effect on the dependent variable. Negative values (in yellow) indicate a favourable reduction in HbA1c towards the first intervention of each comparison (e.g., multicomponent training had a standardised mean HbA1c reduction of 0.63 compared with the control group). Positive values (in blue) indicate an increase in HbA1c towards the first intervention of each comparison

*AER* Aerobic, *BT* Behavioural therapy, *CC* Carbohydrate counting, *CON* Control, *DEd* Diabetes education, *FT* Family therapy, *MC* Multicomponent, *NEd* Nutritional education, *PA* Physical activity, *RT* Resistance training, *SLEd* Sleep education, *SUP* Nutritional supplements

**p value < 0.05

**Fig. 3:**
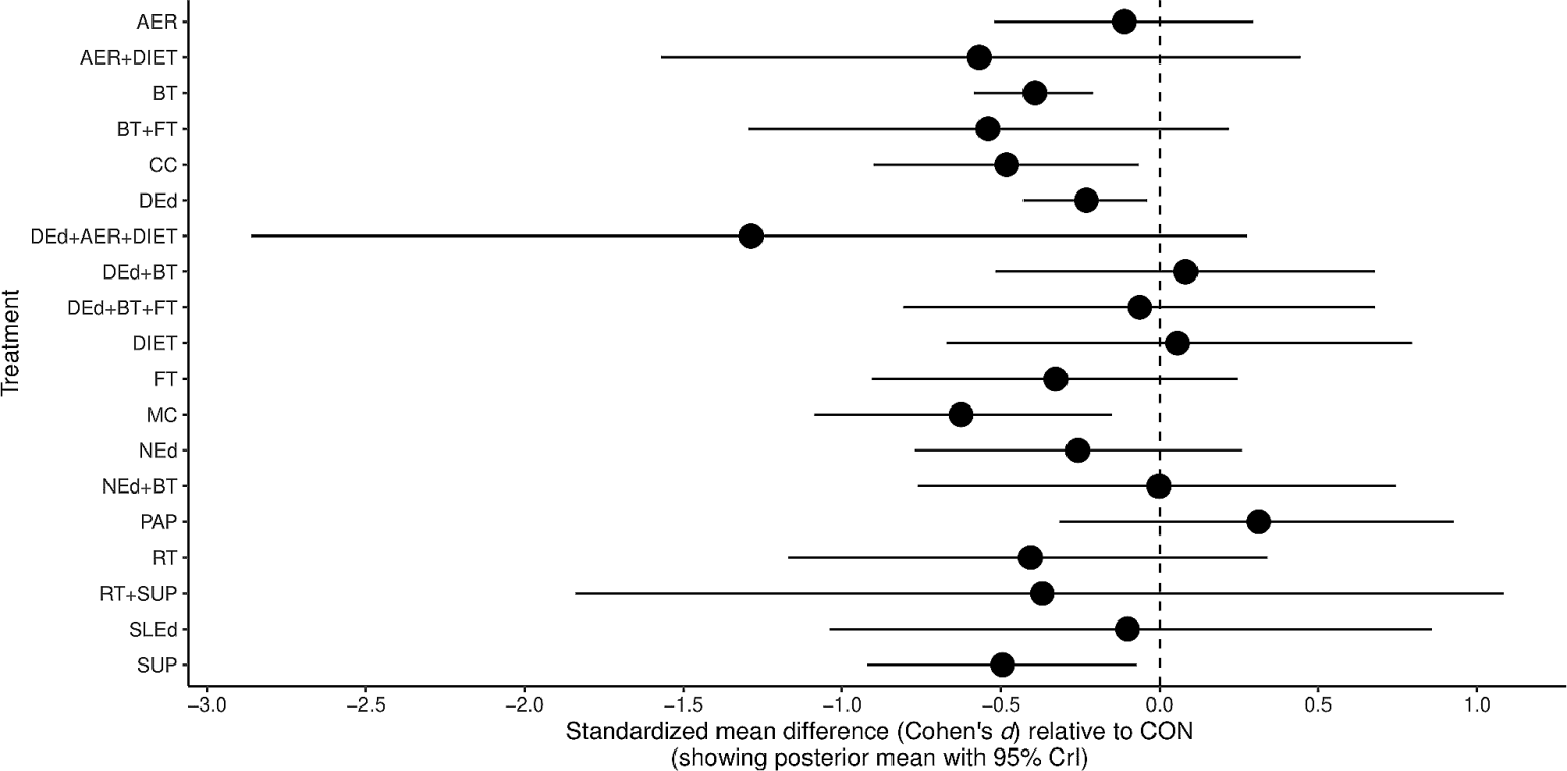
Forest plot displaying results on glycated haemoglobin (HbA1c) levels for various nonpharmacological interventions compared to the control group, which received standard care. Each row represents a specific intervention, while the figure points represent the corresponding standardised mean, and the horizontal lines indicate 95% credible intervals. The model was calculated using a random-effects model. *AER* Aerobic, *BT* Behavioural therapy, *CC* Carbohydrate counting, *CON* Control, *DEd* Diabetes education, *FT* Family therapy, *MC* Multicomponent, *NEd* Nutritional education, *PA* Physical activity, *RT* Resistance training, *SLEd* Sleep Education, *SUP* Nutritional supplements

Regarding HbA1c, almost all interventions showed a greater reduction than controls, although it was non-significant. Instead, increases are reported for physical activity promotion (*n* = 64, SMD = 0.31, 95% CrI –  0.31 to 0.93), diet (*n* = 37, SMD = 0.06, 95% CrI –  0.68 to 0.79), and behavioural therapy accompanied by diabetes education (*n* = 174, SMD = 0.09, 95% CrI –  0.50 to 0.68), exhibiting nonsignificant differences. Conversely, interventions based on multicomponent exercise training (*n* = 214, SMD =– 0.63, 95% CrI – 1.09 to –  0.16), carbohydrate counting (*n* = 173, SMD =– 0.49, 95% CrI – 0.90 to – 0.07), nutritional supplements (*n* = 146, SMD =– 0.49, 95% CrI – 0.92 to – 0.07), behavioural therapy (*n* = 1558, SMD =– 0.39, 95% CrI – 0.58 to – 0.21), and diabetes education (*n* = 1112, SMD =– 0.23, 95% CrI – 0.44 to – 0.04), showed significant reductions in HbA1c (Fig. 3). In addition, ESM Table 6 and ESM Fig. 3, shows the changes in % HbA1c after matching all units of measurement. Interventions based on multicomponent exercise training (*n* = 214, MD =– 0.87, 95% CrI – 1.49 to – 0.23), and behavioural therapy (*n* = 1558, MD =– 0.44, 95% CrI – 0.70 to – 0.20), showed significant reductions in HbA1c.

Concerning the results provided for daily insulin doses requirement, significant effects were observed compared to the control group in interventions involving aerobic training alongside diet (*n* = 10, SMD =– 1.14, 95% CrI – 2.05 to – 0.24), multicomponent training (*n* = 119, SMD =– 0.79, 95% CrI – 1.19 to – 0.34), and nutritional supplements (*n* = 57, SMD = – 0.62, – 1.18 to – 0.12). However, interventions such as resistance strength training with nutritional supplements (*n* = 7, SMD = 1.37, 95% CrI –  0.19 to 2.83), behavioural therapy (*n* = 34, SMD = 0.35, 95% CrI –  0.41 to 1.09), diabetes education (*n* = 137, SMD = 0.24, 95% CrI –  0.13 to 0.58), and carbohydrate counting (*n* = 64, SMD = 0.13, 95% CrI – 0.38 to 0.62) yielded nonsignificant results (ESM Fig. 4).

### Intervention ranking

**Fig. 4:**
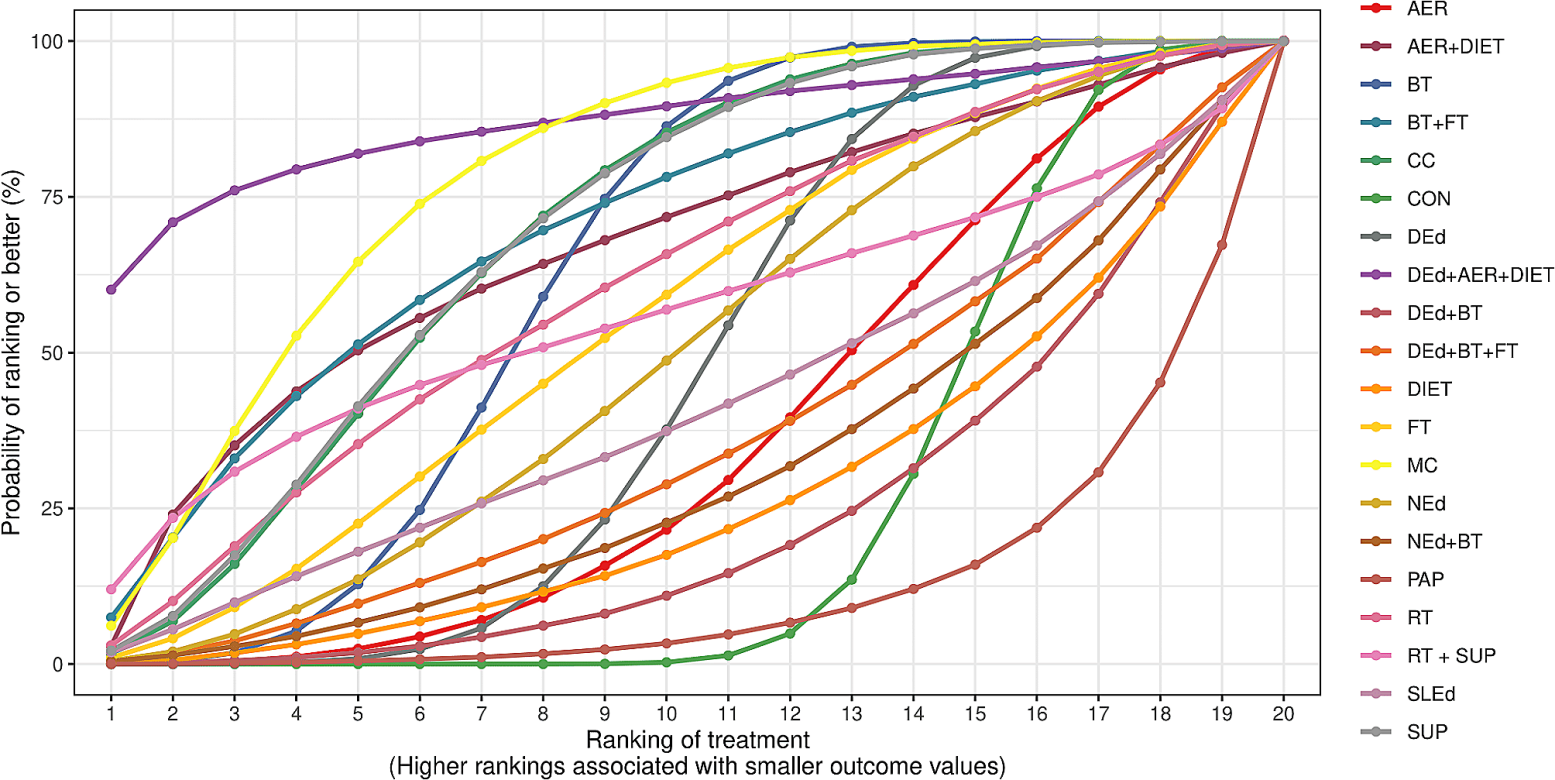
Surface under the cumulative ranking curve (SUCRA) plot illustrating the order of effectiveness on glycated haemoglobin (HbA1c) levels for nonpharmacological interventions based on a network analysis using Bayesian meta-analysis. The position of each intervention on the plot represents its cumulative probability of being classified as the most effective, calculated by the area under the curve. Higher values on the plot indicate a greater probability of effectiveness compared to other interventions. This graph provides clear visual insight into the probabilistic ranking of interventions, aiding in the identification of the most effective options in the context of study. *AER* Aerobic, *BT* Behavioural therapy, *CC* Carbohydrate counting, *CON* Control, *DEd* Diabetes education, *FT* Family therapy, *MC* Multicomponent, *NEd* Nutritional education, *PA* Physical activity, *RT* Resistance training, *SLEd* Sleep education, *SUP* Nutritional supplements

For HbA1c, diabetes education with aerobic training and diet had the highest SUCRA (88.32%), followed by multicomponent exercise (SUCRA = 78.48%) and nutritional supplements (SUCRA = 69.32%) (Fig. 4). For daily insulin dose requirements, aerobic training + diet ranked highest (SUCRA = 94.43%), then multicomponent exercise (SUCRA = 87.3%) and nutritional supplements (SUCRA = 79.84%) (ESM Fig. 5).

### Certainty of the network meta-analysis evidence

The final GRADE report did not show comparisons with a high level of confidence. However, 9 comparisons (4.73%) showed a moderate level of confidence, 102 (53.68%) showed a low level of confidence, and 79 (41.57%) showed a very low level of confidence (ESM Table 9). When examining CINeMA, certain domains related to within-study bias, reporting bias and imprecision raised “some concerns” in most comparisons, with percentages of 84.21%, 99.48% and 56.31%, respectively. On the other hand, the domains of indirectness, heterogeneity and incoherence did not raise concerns in most comparisons, with percentages of 100%, 68.94% and 100%, respectively. However, only 1.5% of comparisons met the threshold for clinically significant HbA1c reductions (SMD ≤– 0.8).

### Additional analyses

Regarding the meta-regressions conducted for all nonpharmacological interventions versus the control group, significant associations were observed between participants' age (unstandardised beta coefficient [*B*] =– 0.019, 95% CrI – 0.027 to – 0.011), intervention duration (*B* =– 0.007, 95% CrI – 0.010 to – 0.004), and the duration of diabetes since diagnosis (*B* =– 0.038, 95% CrI – 0.056 to –  0.020), in relation to HbA1c (ESM Table 7). When considering comparisons that included more than 10 studies, similar moderating effects were found only in the comparison between behavioural therapy versus control. While in the comparison between diabetes education versus control, no moderating effect of these variables on the estimates was observed (ESM Table 8).

On the other hand, Bayesian funnel plots in ESM Fig. 6 show the publication bias assessment of all non-pharmacological interventions versus control, and of those comparisons with more than 10 studies (behavioural therapy vs. control, and diabetes education vs. control). Overall, funnel plots suggest a predominantly symmetric distribution of study results around the pooled effect size, with only slight asymmetry indicating minimal publication bias.

## Discussion

This ground-breaking NMA represents a comprehensive assessment of nonpharmacological interventions aimed at enhancing glycaemic control in paediatric type 1 diabetes. Noteworthy is the discernible reduction in HbA1c levels and daily insulin requirements due to various non-pharmacological interventions. In particular, the incorporation of multicomponent exercise (i.e., resistance exercises alongside aerobic training) have contributed substantially to reductions of up to 0.87% in HbA1c levels. These findings underscore the pivotal role of structured physical activity in the management of type 1 diabetes in the paediatric and adolescent population. The significance lies not only in the overall efficacy of these interventions but also in their potential to tailor diabetes management strategies for young individuals, emphasising the need for a holistic approach beyond pharmacological measures. Implementing these evidence-based strategies could pave the way for optimizing glycaemic regulation, thereby fostering better health outcomes and improved quality of life for children and adolescents living with type 1 diabetes. The study also aligns with ADA guidelines showing benefits of interventions like carbohydrate counting, behavioural therapy, and diabetes education [43].

A growing body of research recognises the influence of exercise on metabolic control in youth with type 1 diabetes [11, 43, 44]. Our results concur with a prior meta-analysis underscoring the value of concurrent exercise combining aerobic and resistance modalities [13]. Another study in youth with overweight/obesity found greater benefits from diverse, multicomponent exercises on insulin markers [45], reinforcing the importance of multifaceted physical training for glycaemic regulation across populations. Moreover, we observed reduced insulin requirements with multicomponent exercise, supported by other studies [46, 47] and the aforementioned meta-analysis [13]. The proposed mechanisms are associated with exercise-induced enhancements in glucose uptake, insulin sensitivity, and related metabolic factors [48–52].

Our analysis also provides evidence that certain nutritional supplements like probiotics, cinnamon, antioxidants, honey, and camel milk significantly lower HbA1c. However, these findings should be interpreted cautiously given the limited supplement studies. Indeed, research involving probiotic-based supplementation has revealed reductions in HbA1c levels and insulin requirements among diabetic populations [53, 54], possibly by modulating carbohydrate absorption and influencing glucose regulation through the production of short-chain fatty acids [55, 56]. Likewise, honey-based supplements may improve glycaemic control due to its fructose content, which can facilitate glucose metabolism and stimulate insulin release [57, 58].

On the other hand, meta-regression analyses showed that, overall, the higher the age, the longer the duration of the intervention and the longer the time suffering from type 1 diabetes, the greater the reductions obtained. Specifically, for behavioural therapy, older ager, longer intervention and disease duration were associated with greater reductions obtained. These results are in line with some studies suggesting that prolonged interventions over time are more efficient in controlling metabolic parameters in individuals with diabetes [13, 59]. However, the specific influence of mean age or disease duration on nonpharmacological interventions is limited. Given that we were only able to perform specific meta-regressions for some interventions (i.e., behavioural therapy, diabetes education), further studies in interventions with 10 or fewer studies are required to draw stronger conclusions.

This study has several strengths, including the ability of NMA to precisely quantify and rank intervention effects to inform clinical translation. However, limitations warrant consideration when interpreting the results. The role of mean age, intervention duration and duration of the disease observed across studies may influence the outcomes of the different groups or nodes formed within this NMA. Additionally, using CINeMA, many comparisons were rated low/very low quality, restricting result generalisability. While moderate effects were observed, few comparisons showed clinically meaningful HbA1c changes. Grouping heterogeneous interventions limits determining the most effective individual approaches. Furthermore, diabetes management differences across studies were not addressed. Nonetheless, this NMA signposts future high-quality research on multidisciplinary, tailored interventions for youth with type 1 diabetes.

In conclusion, despite the generally low certainty of evidence and the need to interpret the results with caution, the findings seem to emphasize the potential of a comprehensive management approach for improving glycaemic control in children and adolescents with type 1 diabetes. This approach includes integrating multicomponent exercise and individualized nutritional supplementation to lower HbA1c levels, reduce insulin requirements, and enhance overall health. These results underscore the value of various holistic regimens aimed at reducing glycaemic control and facilitating the management of type 1 diabetes mellitus in young individuals. It is suggested that the choice of intervention types be tailored to the specific needs of patients, offering a promising model for more effective and complementary care alongside traditional treatment. However, further research is needed to confirm optimal protocols and combinations in this approach.

### Supplementary Information

Below is the link to the electronic supplementary material.

Supplementary material 1 (DOC 1359 KB)

## Data Availability

No datasets were generated or analysed during the current study.
