# Supplementary material for: Nonpharmacological interventions on glycated haemoglobin in youth with type 1 diabetes: a Bayesian network meta-analysis
Source: Cardiovasc Diabetol. 2024 Jul 1;23:230. doi: 10.1186/s12933-024-02301-3 (PMC11218128; doi:10.1186/s12933-024-02301-3)
Supplement: Supplementary file 1 — Supplementary material 1 (DOC 1359 KB) [file 12933_2024_2301_MOESM1_ESM.doc]

**Nonpharmacological interventions and HbA1c in youth with type 1 diabetes: A Bayesian network meta-analysis**

*Muñoz-Pardeza et al.*

**Electronic supplementary material**

**ESM Table 1.** Search formula for electronic database.

**ESM Table 2.** Explanation of the different categories of the study.

**ESM Table 3.** Excluded studies and reasons for exclusion.

**ESM Table 4.** Characteristics of the included studies.

**ESM Method 1.** Characteristics of the included studies.

**ESM Table 5:** League table of the network meta-analysis for daily insulin dose requirement.

**ESM Table 6:** Complementary league table from the network meta-analysis for changes in % glycated haemoglobin.

**ESM Table 7.** Meta-regression analyses by participants’ age, number of girls, intervention duration, and disease duration, for all interventions (only versus control) included in the network meta-analysis.

**ESM Table 8.** Meta-regression analyses by participants’ age, number of girls, intervention duration, and disease duration, for those intervention types with 10 or more studies.

**ESM Table 9.** Confidence in Network Meta-Analysis (CINeMA) confidence rating.

**ESM Figure 1.** Risk of bias results (RoB 2).

**ESM Figure 2.** Network plot of available comparisons between different non-pharmacological interventions on insulin doses in children and adolescents with type 1 diabetes mellitus. The size of the nodes is directly proportional to the number of participants randomly assigned to each intervention. The width of the connecting lines corresponds to the number of studies comparing the respective interventions.

**ESM Figure 3.** Forest plot showing results on % HbA1c after various non-pharmacological interventions compared to the control group, which received standard care. Each row represents a specific intervention, while the dots in the figure represent the change in % HbA1c, and the horizontal lines indicate 95% credible intervals. The model was estimated using a random effects model.

**ESM Figure 4.** Forest plot displaying results on daily insulin doses requirement for various non-pharmacological interventions compared to the control group, which received standard care. Each row represents a specific intervention, while the figure points represent the corresponding standardised mean, and the horizontal lines indicate 95% credible intervals. The model was calculated using a random-effects model.

**ESM Figure 5.** Rankogram for daily insulin doses requirement.

**ESM Figure 6.** Bayesian funnel-plots for reporting bias for all nonpharmacological interventions and those comparisons with more than 10 studies.

**ESM References**

**ESM Table 1.**  Search formula for electronic database.

| **PubMed (*n* = 291)** |
| --- |
| (("Physical Fitness"[Mesh] OR "Aerobic Fitness" OR "Aerobic Capacity" OR "Maximal Oxygen Consumption" OR "Muscle Strength"[Mesh] OR "Exercise"[Mesh] OR "Exercise Therapy"[Mesh] OR "Sports"[Mesh] OR "Sedentary Behavior"[Mesh]) OR ("Diet"[Mesh] OR “Atkins” OR “Dukan” OR “DASH” OR “South Beach” OR “Ornish” OR “Weight Watcher” OR “Carbohydrate Restricted” OR “Low Carb” OR “Fat Restricted” OR “Low Fat” OR “Supplement*” OR “Prebiotic” OR “Probiotic” OR “Nutritional Education” OR “Carb Count”) OR ("Psychology" OR "Positive Psychology" OR "Motivation" OR "Motivational" OR "Motivational Interviewing" OR "Satisfaction" OR "Communication" OR "Education" OR "Behavioral" OR "Cognitive Behaviour" OR "Conductual" OR "Psychosocial" OR "Text messag*" OR "Mobile Health" OR "eHealth" OR "Meditation" OR "Mindfulness")) AND ("Diabetes Mellitus, Type 1"[Mesh]) AND ("glycosylated haemoglobin" OR "HbA1c") AND ("Preschool*" OR "child*" OR "adolescen*" OR "teen*" OR "youth*" OR "young*")) |
| **Web Of Science (*n* = 1046)** |
| (TS=("Physical Fitness") OR TS=("Cardiorespiratory Fitness") OR TS=("Aerobic Fitness") OR TS=("Aerobic Capacity") OR TS=("Maximal Oxygen Consumption") OR TS=("Muscle Strength") OR TS=("Exercise") OR TS=("Physical Activity") OR TS=("Exercise Therapy") OR TS=("Sports") OR TS=("Sedentary Behavior")) OR (TS=("Diet*") OR TS=("Atkins") OR TS=("Dukan") OR TS=("Mediterranean Diet") OR TS=("Paleo Diet") OR TS=("Paleolithic Diet") OR TS=("South Beach") OR TS=("DASH") OR TS=("Ornish") OR TS=("Weight Watcher") OR TS=("Western") OR TS=("Vegetarian Diet") OR TS=("Supplement") OR TS=("Prebiotic") OR TS=("Probiotic") OR TS=("Carbohydrate Restricted") OR TS=("Low Carb") OR TS=("Fat Restricted") OR TS=("Low Fat") OR TS=("Carb Count") OR TS=("Nutritional Education") OR TS=("Caloric Restriction")) OR (TS=("Psychology") OR TS=("Positive Psychology") OR TS=("Motivation") OR TS=("Motivational") OR TS=("Motivational Interviewing") OR TS=("Satisfaction") OR TS=("Communication") OR TS=("Education") OR TS=("Behavioral") OR TS=("Cognitive Behaviour") OR TS=("Conductual") OR TS=("Psychosocial") OR TS=("Text messag*") OR TS=("Mobile Health") OR TS=("eHealth") OR TS=("Meditation") OR TS=("Mindfulness")) AND (TS=("Diabetes Mellitus, Type 1") OR TS=("Type 1 Diabetes")) AND (TS=("HbA1C") OR TS=("glycosylated haemoglobin")) AND (TS=("RCT") OR TS=("Randomized Controlled Trial")) AND (TS=("Preschool") OR TS=("Child*") OR TS=("adolescen*") OR TS=("youth") OR TS=("young*")) |
| **Sport Discus (*n* = 86)** |
| (“Exercise” OR “Physical Activity” OR “Diet” OR “Nutritional Education” OR “motivational interviewing” OR “cognitive behavioral therapy”) AND (“diabetes mellitus type 1”) AND (“hba1c”) AND (“randomized controlled trials”) AND (“children” OR “adolescents”) |
| **Scopus (*n* = 2374)** |
| ( TITLE-ABS-KEY ( "Physical Fitness" ) OR TITLE-ABS-KEY ( "Aerobic Fitness" ) OR TITLE-ABS-KEY ( "Aerobic Capacity" ) OR TITLE-ABS-KEY ( "Maximal Oxygen Consumption" ) OR TITLE-ABS-KEY ( "Muscle Strength" ) OR TITLE-ABS-KEY ( "Exercise" ) OR TITLE-ABS-KEY ( "Exercise Therapy" ) OR TITLE-ABS-KEY ( "Sports" ) OR TITLE-ABS-KEY ( "Sedentary Behavior" ) OR TITLE-ABS-KEY ( "Diet" ) OR TITLE-ABS-KEY ( "Atkins" ) OR TITLE-ABS-KEY ( "Dukan" ) OR TITLE-ABS-KEY ( "Mediterranean Diet" ) OR TITLE-ABS-KEY ( "Paleo Diet" ) OR TITLE-ABS-KEY ( "Paleolithic Diet" ) OR TITLE-ABS-KEY ( "South Beach" ) OR TITLE-ABS-KEY ( "DASH" ) OR TITLE-ABS-KEY ( "Ornish" ) OR TITLE-ABS-KEY ( "Weight Watcher" ) OR TITLE-ABS-KEY ( "Western" ) OR TITLE-ABS-KEY ( "Vegetarian Diet" ) OR TITLE-ABS-KEY ( "Supplement" ) OR TITLE-ABS-KEY ( "Prebiotic" ) OR TITLE-ABS-KEY ( "Probiotic" ) OR TITLE-ABS-KEY ( "Carbohydrate Restricted" ) OR TITLE-ABS-KEY ( "Low Carb" ) OR TITLE-ABS-KEY ( "Fat Restricted" ) OR TITLE-ABS-KEY ( "Low Fat" ) OR TITLE-ABS-KEY ( "Carb Count" ) OR TITLE-ABS-KEY ( "Nutritional Education" ) OR TITLE-ABS-KEY ( "Caloric Restriction" ) OR TITLE-ABS-KEY ( "Psychology" ) OR TITLE-ABS-KEY ( "Positive Psychology" ) OR TITLE-ABS-KEY ( "Motivation" ) OR TITLE-ABS-KEY ( "Motivational" ) OR TITLE-ABS-KEY ( "Motivational Interviewing" ) OR TITLE-ABS-KEY ( "Satisfaction" ) OR TITLE-ABS-KEY ( "Communication" ) OR TITLE-ABS-KEY ( "Education" ) OR TITLE-ABS-KEY ( "Behavioral" ) OR TITLE-ABS-KEY ( "Cognitive Behaviour" ) OR TITLE-ABS-KEY ( "Conductual" ) OR TITLE-ABS-KEY ( "Psychosocial" ) OR TITLE-ABS-KEY ( "Text messag*" ) OR TITLE-ABS-KEY ( "Mobile Health" ) OR TITLE-ABS-KEY ( "eHealth" ) OR TITLE-ABS-KEY ( "Meditation" ) OR TITLE-ABS-KEY ( "Mindfulness" ) ) AND ( TITLE-ABS-KEY ( "Diabetes Mellitus, Type 1" ) OR TITLE-ABS-KEY ( "Type 1 Diabetes" ) ) AND ( TITLE-ABS-KEY ( "HbA1C" ) OR TITLE-ABS-KEY ( "glycosylated haemoglobin" ) ) AND ( LIMIT-TO ( EXACTKEYWORD , "Adolescent" ) OR LIMIT-TO ( EXACTKEYWORD , "Child" ) OR LIMIT-TO ( EXACTKEYWORD , "Clinical Trial" ) OR LIMIT-TO ( EXACTKEYWORD , "Randomized Controlled Trial" ) OR LIMIT-TO ( EXACTKEYWORD , "Children" ) OR LIMIT-TO ( EXACTKEYWORD , "Controlled Clinical Trial" ) ) OR LIMIT-TO ( DOCTYPE , "ar" ) ) |

**ESM Table 2.** Explanation of the different categories of the study.

| ***Control*** (CON) |
| --- |
| Study groups that have received usual care or minimal recommendations in order not to intervene in the daily lives of the participants. |
| ***Aerobic exercise training*** (AER) |
| Structured physical exercise focused on aerobic activities in order to increase cardiorespiratory capacity. |
| ***Behavioural Therapy*** (BT) |
| Therapy based on behaviour modification through techniques derived from behavioural psychology, using change strategies based on learning and the environment. |
| ***Carbohydrate counting*** (CC) |
| Technique based on the calculation of the amount of carbohydrates in the foods consumed to control blood glucose levels. |
| ***Diabetic education*** (DEd) |
| Interventions based on providing proportionate knowledge, skills, and/or innovative tools necessary for the management of diabetes mellitus. |
| ***Diet*** (DIET) |
| Interventions based on modifying daily food intake from various perspectives or purposes. |
| ***Family therapy*** (FT) |
| Interventions based on family involvement aimed at assisting in the management of diabetes mellitus. |
| ***Multicomponent exercise training*** (MC) |
| Structured physical exercise that combines two or more physical activities in order to improve various physical fitness abilities. This group includes concurrent training, a specific type of training that combines aerobic and resistance training. |
| ***Nutritional education*** (NEd) |
| Interventions based on educating about specific aspects of diet and nutrients, as well as the relationship between these and diabetes mellitus. |
| ***Physical activity promotion*** (PAP) |
| Interventions focused on promoting unstructured physical activity to foster healthy habits in individuals with diabetes mellitus. |
| ***Resistance exercise training*** (RT) |
| Structured physical exercise focused on strengthening activities in order to increase muscle strength. |
| ***Sleep education*** (SLEd) |
| Interventions based on educating about sleep habits to promote improvement in the management of diabetes mellitus. |
| ***Nutritional supplements*** (SUP) |
| Interventions based on analyzing the effect of different supplements on the metabolic control of diabetes mellitus (Most interventions in this group include supplements based on probiotic compounds). |

**ESM Table 3.** Excluded studies and reasons for exclusion.

| **Reason for exclusion: No randomized controlled trial (RCT)** |
| --- |
| Alvarado-Martel, D., Boronat, M., Alberiche-Ruano, M. D. P., Algara-González, M. A., Ramallo-Fariña, Y., & Wägner, A. M. (2020). Motivational Interviewing and Self-Care in Type 1 Diabetes: A Randomized Controlled Clinical Trial Study Protocol. *Frontiers in Endocrinology*, *11*, 574312. https://doi.org/10.3389/fendo.2020.574312  Aouadi, R., Khalifa, R., Aouidet, A., Mansour, A. B., Rayana, M. C. B., Mdini, F., Bahri, S., & Stratton, G. (2011). Aerobic training programs and glycemic control in diabetic children in relation to exercise frequency. *Journal of Sports Medicine and Physical Fitness*, *51*(3), 393–400. Scopus.  Ash, G. I., Joiner, K. L., Savoye, M., Baker, J. S., Gerosa, J., Kleck, E., Patel, N. S., Sadler, L. S., Stults‐Kolehmainen, M., Weinzimer, S. A., & Grey, M. (2019). Feasibility and safety of a group physical activity program for youth with type 1 diabetes. *Pediatric Diabetes*, *20*(4), 450–459. https://doi.org/10.1111/pedi.12841  Barnes, T. L., Crandell, J. L., Bell, R. A., Mayer-Davis, E. J., Dabelea, D., & Liese, A. D. (2013). Change in DASH diet score and cardiovascular risk factors in youth with type 1 and type 2 diabetes mellitus: The search for diabetes in youth study. *Nutrition and Diabetes*, *3*(OCTOBER). Scopus. https://doi.org/10.1038/nutd.2013.32  Benslama, Y., Dennouni-Medjati, N., Dali-Sahi, M., Kahloula, S., Behar, A., Kachekouche, Y., Meziane, F. Z., & Harek, Y. (2020). Dietary intake of magnesium in a type 1 diabetic pediatric population. *Magnesium Research*, *33*(2), 37–44. Scopus. https://doi.org/10.1684/mrh.2020.0467  Bin-Abbas, B., Jabbari, M., Al-Fares, A., El-Dali, A., & Al-Orifi, F. (2014). Effect of mobile phone short text messages on glycaemic control in children with type 1 diabetes. *Journal of Telemedicine and Telecare*, *20*(3), 153–156. Scopus. https://doi.org/10.1177/1357633X14529244  Blake, H., Quirk, H., Leighton, P., Randell, T., Greening, J., Guo, B., & Glazebrook, C. (2016). Feasibility of an online intervention (STAK-D) to promote physical activity in children with type 1 diabetes: Protocol for a randomised controlled trial. *Trials*, *17*(1), 583. https://doi.org/10.1186/s13063-016-1719-0  Cadario, F., Prodam, F., Pasqualicchio, S., Bellone, S., Bonsignori, I., Demarchi, I., Monzani, A., & Bona, G. (n.d.). *Lipid profile and nutritional intake in children and adolescents with Type 1 diabetes improve after a structured dietician training to a Mediterranean-style diet*.  Cafazzo, J. A., Casselman, M., Hamming, N., Katzman, D. K., & Palmert, M. R. (2012). Design of an mHealth App for the Self-management of Adolescent Type 1 Diabetes: A Pilot Study. *Journal of Medical Internet Research*, *14*(3), e70. https://doi.org/10.2196/jmir.2058  Choi, Y. S., Berry-Cabán, C., & Nance, J. (2013). Telemedicine in Paediatric Patients with Poorly Controlled Type 1 Diabetes. *Journal of Telemedicine and Telecare*, *19*(4), 219–221. https://doi.org/10.1258/jtt.2012.120506  Clarke, J., Vatiliotis, V., Verge, C. F., Holmes-Walker, J., Campbell, L. V., Wilhelm, K., & Proudfoot, J. (2015). A Mobile Phone and Web-Based Intervention for Improving Mental Well-Being in Young People With Type 1 Diabetes: Design of a Randomized Controlled Trial. *JMIR Research Protocols*, *4*(2), e50. https://doi.org/10.2196/resprot.4032  Deda, L., Yeshayahu, Y., Sud, S., Cuerden, M., Cherney, D. Z. I., Sochett, E. B., & Mahmud, F. H. (2018). Improvements in peripheral vascular function with vitamin D treatment in deficient adolescents with type 1 diabetes. *PEDIATRIC DIABETES*, *19*(3), 457–463. https://doi.org/10.1111/pedi.12595  Delahanty, L. M., Nathan, D. M., Lachin, J. M., Hu, F. B., Cleary, P. A., Ziegler, G. K., Wylie-Rosett, J., & Wexler, D. J. (2009). Association of diet with glycated haemoglobin during intensive treatment of type 1 diabetes in the Diabetes Control and Complications Trial. *The American Journal of Clinical Nutrition*, *89*(2), 518–524. https://doi.org/10.3945/ajcn.2008.26498  Döğer, E., Bozbulut, R., Şebnem Soysal Acar, A., Ercan, Ş., Uğurlu, A. K., Akbaş, E. D., Bideci, A., Çamurdan, O., & Cinaz, P. (2019). Effect of telehealth system on glycemic control in children and adolescents with type 1 diabetes. *JCRPE Journal of Clinical Research in Pediatric Endocrinology*, *11*(1), 70–75. Scopus. https://doi.org/10.4274/jcrpe.galenos.2018.2018.0017  Frielitz, F.-S., Müller-Godeffroy, E., Hübner, J., Eisemann, N., Dördelmann, J., Menrath, I., Katalinic, A., Hiort, O., & von Sengbusch, S. (2020). Monthly Video-Consultation for Children With Type 1 Diabetes Using a Continuous Glucose Monitoring System: Design of ViDiKi, a Multimethod Intervention Study to Evaluate the Benefit of Telemedicine. *Journal of Diabetes Science and Technology*, *14*(1), 105–111. Scopus. https://doi.org/10.1177/1932296819861991  Froisland, D. H., & Arsand, E. (2015). Integrating visual dietary documentation in mobile-phone-based self-management application for adolescents with type 1 diabetes. *Journal of Diabetes Science and Technology*, *9*(3), 541–548. Scopus. https://doi.org/10.1177/1932296815576956  García-Pérez, L., Perestelo-Pérez, L., Serrano-Aguilar, P., & Trujillo-Martín, M. D. M. (2010). Effectiveness of a Psychoeducative Intervention in a Summer Camp for Children With Type 1 Diabetes Mellitus. *The Diabetes Educator*, *36*(2), 310–317. https://doi.org/10.1177/0145721710361784  Guttmann-Bauman, I., Kono, J., Lin, A. L., Ramsey, K. L., & Boston, B. A. (2018). Use of Telehealth Videoconferencing in Pediatric Type 1 Diabetes in Oregon. *Telemedicine and E-Health*, *24*(1), 86–88. Scopus. https://doi.org/10.1089/tmj.2017.0072  Haas, J., Persson, M., Brorsson, A. L., Toft, E. H., & Olinder, A. L. (2017). Guided self-determination-young versus standard care in the treatment of young females with type 1 diabetes: Study protocol for a multicentre randomized controlled trial. *Trials*, *18*(1), 562. https://doi.org/10.1186/s13063-017-2296-6  Haider, D. G., Pleiner, J., Francesconi, M., Wiesinger, G. F., Müller, M., & Wolzt, M. (2006). Exercise training lowers plasma visfatin concentrations in patients with type 1 diabetes. *The Journal of Clinical Endocrinology and Metabolism*, *91*(11), 4702–4704. https://doi.org/10.1210/jc.2006-1013  Haline Dalsgaard, C. S., Patrícia de C. Padilha, Jorge Luiz Luescher, Renata Szu,-. (2014). Glycemic control and lipid profile of children and adolescents undergoing two different dietetic treatments for type 1 diabetes mellitus. *NUTRICION HOSPITALARIA*, *3*, 547–552. https://doi.org/10.3305/nh.2014.29.3.7116  Harmer, A. R., Chisholm, D. J., McKenna, M. J., Hunter, S. K., Ruell, P. A., Naylor, J. M., Maxwell, L. J., & Flack, J. R. (2008). Sprint training increases muscle oxidative metabolism during high-intensity exercise in patients with type 1 diabetes. *Diabetes Care*, *31*(11), 2097–2102. https://doi.org/10.2337/dc08-0329  Hawkes, C. P., Willi, S. M., & Murphy, K. M. (2019). A structured 1-year education program for children with newly diagnosed type 1 diabetes improves early glycemic control. *Pediatric Diabetes*, *20*(4), 460–467. Scopus. https://doi.org/10.1111/pedi.12849  Ho, J., Nicolucci, A. C., Virtanen, H., Schick, A., Meddings, J., Reimer, R. A., & Huang, C. (2019). Effect of Prebiotic on Microbiota, Intestinal Permeability, and Glycemic Control in Children with Type 1 Diabetes. *Journal of Clinical Endocrinology and Metabolism*, *104*(10), 4427–4440. Scopus. https://doi.org/10.1210/jc.2019-00481  Iafusco, D., Galderisi, A., Nocerino, I., Cocca, A., Zuccotti, G., Prisco, F., & Scaramuzza, A. (2011). Chat Line for Adolescents with Type 1 Diabetes: A Useful Tool to Improve Coping with Diabetes: A 2-Year Follow-Up Study. *Diabetes Technology & Therapeutics*, *13*(5), 551–555. https://doi.org/10.1089/dia.2010.0188  Joubert, M., Armand, C., Morera, J., Tokayeva, L., Guillaume, A., & Reznik, Y. (2016). Impact of a serious videogame designed for flexible insulin therapy on the knowledge and behaviors of children with type 1 diabetes: The LUDIDIAB pilot study. *Diabetes Technology and Therapeutics*, *18*(2), 52–58. Scopus. https://doi.org/10.1089/dia.2015.0227  Lansing, A. H., Stanger, C., Budney, A., Christiano, A. S., & Casella, S. J. (2016). Pilot Study of a Web-Delivered Multicomponent Intervention for Rural Teens with Poorly Controlled Type 1 Diabetes. *Journal of Diabetes Research*, *2016*, 1–8. https://doi.org/10.1155/2016/7485613  Maffeis, C., Morandi, A., Ventura, E., Sabbion, A., Contreas, G., Tomasselli, F., Tommasi, M., Fasan, I., Costantini, S., & Pinelli, L. (2012). Diet, physical, and biochemical characteristics of children and adolescents with type 1 diabetes: Relationship between dietary fat and glucose control. *Pediatric Diabetes*, *13*(2), 137–146. https://doi.org/10.1111/j.1399-5448.2011.00781.x  Marigliano, M., Morandi, A., Maschio, M., Sabbion, A., Contreas, G., Tomasselli, F., Tommasi, M., & Maffeis, C. (2013). Nutritional education and carbohydrate counting in children with type 1 diabetes treated with continuous subcutaneous insulin infusion: The effects on dietary habits, body composition and glycometabolic control. *Acta Diabetologica*, *50*(6), 959–964. Scopus. https://doi.org/10.1007/s00592-013-0491-9  Martyn-Nemeth, P., Duffecy, J., Quinn, L., Reutrakul, S., Steffen, A. D., Burke, L., Clark Withington, M. H., Irsheed, G. A., Perez, R., Park, M., Saleh, A., Mihailescu, D., & Baron, K. G. (2022). Sleep optimization to improve glycemic control in adults with type 1 diabetes: Study protocol for a randomized controlled parallel intervention trial. *Trials*, *23*(1). Scopus. https://doi.org/10.1186/s13063-022-06565-6  Mirabelli, M., Chiefari, E., Puccio, L., Foti, D. P., & Brunetti, A. (2020). Potential Benefits and Harms of Novel Antidiabetic Drugs During COVID-19 Crisis. *International Journal of Environmental Research and Public Health*, *17*(10), 3664. https://doi.org/10.3390/ijerph17103664  Mosher, P., Nash, M., Perry, A., LaPerriere, A., & Goldberg, R. (1998). Aerobic circuit exercise training: Effect on adolescents with well-controlled insulin-dependent diabetes mellitus. A*rchives of Physical Medicine and Rehabilitation*, *79*(6), 652–657. https://doi.org/10.1016/S0003-9993(98)90039-9  Neuman, V., Pruhova, S., Kulich, M., Kolouskova, S., Vosahlo, J., Romanova, M., Petruzelkova, L., Obermannova, B., Funda, D. P., Cinek, O., & Sumnik, Z. (2020). Gluten-free diet in children with recent-onset type 1 diabetes: A 12-month intervention trial. *Diabetes, Obesity and Metabolism*, *22*(5), 866–872. Scopus. https://doi.org/10.1111/dom.13974  Nwosu, B. U., & Maranda, L. (2014). The effects of vitamin D supplementation on hepatic dysfunction, vitamin D status, and glycemic control in children and adolescents with vitamin D deficiency and either type 1 or type 2 diabetes mellitus. *PLoS ONE*, *9*(6). Scopus. https://doi.org/10.1371/journal.pone.0099646  Paganus, A., Mäenpää, J., Åkerblom, H. K., Stenman, U.-H., Knip, M., & Simell, O. (1987). Beneficial Effects of Palatable Guar and Guar Plus Fructose Diets in Diabetic Children. *Acta Paediatrica*, *76*(1), 76–81. https://doi.org/10.1111/j.1651-2227.1987.tb10418.x  Peña, N. V., Torres, M., Cardona, J. A. C., & Iniesta, R. (2013). Impact of telemedicine assessment on glycemic variability in children with type 1 diabetes mellitus. *Diabetes Technology and Therapeutics*, *15*(2), 136–142. Scopus. https://doi.org/10.1089/dia.2012.0243  Pramanik, B. K., Angelin, J. J., Mathai, V. J., Mathai, S., Korula, S., & Simon, A. (2019). Smartphone App as Motivational Intervention to Improve Glycemic Control in Adolescents with Type 1 Diabetes. *The Indian Journal of Pediatrics*, *86*(12), 1118–1123. https://doi.org/10.1007/s12098-019-03035-x  Savilahti, E., Härkönen, T., Savilahti, E. M., Kukkonen, K., Kuitunen, M., & Knip, M. (2018). Probiotic intervention in infancy is not associated with development of beta cell autoimmunity and type 1 diabetes. *Diabetologia*, *61*(12), 2668–2670. https://doi.org/10.1007/s00125-018-4738-4  Schwingshandl, J., Rippel, S., Unterluggauer, M., & Borkenstein, M. (1994). Effect of the introduction of dietary sucrose on metabolic control in children and adolescents with type I diabetes. *Acta Diabetologica*, *31*(4), 205–209. https://doi.org/10.1007/BF00571952  Seeger, J. P. H., Thijssen, D. H. J., Noordam, K., Cranen, M. E. C., Hopman, M. T. E., & Nijhuis-van der Sanden, M. W. G. (2011). Exercise training improves physical fitness and vascular function in children with type 1 diabetes. *Diabetes, Obesity & Metabolism*, *13*(4), 382–384. https://doi.org/10.1111/j.1463-1326.2011.01361.x  Suh, J., Choi, H. S., Kwon, A., Chae, H. W., Eom, S., & Kim, H.-S. (2019). Once-weekly supervised combined training improves neurocognitive and psychobehavioral outcomes in young patients with type 1 diabetes mellitus. *Journal of Pediatric Endocrinology and Metabolism*. Scopus. https://doi.org/10.1515/jpem-2019-0120  von Sengbusch, S., Schneidewind, J., Bokelmann, J., Scheffler, N., Bertram, B., Frielitz, F.-S., Hiort, O., & Lange, K. (2022). Monthly video consultation for children and adolescents with type 1 diabetes mellitus during the COVID-19 pandemic. *Diabetes Research and Clinical Practice*, *193*. Scopus. https://doi.org/10.1016/j.diabres.2022.110135  Vyas, C., Dalal, L., Talaviya, P., & Saboo, B. (2017). Multiple educational programs improves glycemic control, quality of life with diminishing the impact of diabetes in poorly controlled type 1 diabetics. *Diabetes and Metabolic Syndrome: Clinical Research and Reviews*, *11*, S601–S606. Scopus. https://doi.org/10.1016/j.dsx.2017.04.011  Weissberg-Benchell, J., Rausch, J., Iturralde, E., Jedraszko, A., & Hood, K. (2016). A randomized clinical trial aimed at preventing poor psychosocial and glycemic outcomes in teens with type 1 diabetes (T1D). *CONTEMPORARY CLINICAL TRIALS*, *49*, 78–84. https://doi.org/10.1016/j.cct.2016.05.006 |
| **Reason for exclusion: Adults** |
| Battista, M.-C., Labonté, M., Ménard, J., Jean-Denis, F., Houde, G., Ardilouze, J.-L., & Perron, P. (2012). Dietitian-coached management in combination with annual endocrinologist follow up improves global metabolic and cardiovascular health in diabetic participants after 24 months. *Applied Physiology, Nutrition and Metabolism*, *37*(4), 610–620. Scopus. https://doi.org/10.1139/H2012-025  Cohen, D., Dodds, R., & Viberti, G. (1987). *Effect of protein restriction in insulin dependent diabetics at risk of nephropathy*. *294*.  De Melo, F. T. C., Felício, K. M., De Queiroz, N. N. M., De Rider Brito, H. A., Neto, J. F. A., Janaú, L. C., De Souza Neto, N. J. K., Silva, A. L. A., De Lemos, M. N., De Oliveira, M. C. N. I., De Alcântara, A. L., De Moraes, L. V., De Souza, A. J. A., Said, N. M., Da Silva, W. M., De Lemos, G. N., Dos Santos, M. C., Albuquerque Silva, L. D. S. D., Motta, A. R. B., … Felício, J. S. (2022). High-dose Vitamin D Supplementation on Type 1 Diabetes Mellitus Patients: Is there an Improvement in Glycemic Control? *Current Diabetes Reviews*, *18*(1). Scopus. https://doi.org/10.2174/1573399817666210106102643  Drion, I., Pameijer, L. R., van Dijk, P. R., Groenier, K. H., Kleefstra, N., & Bilo, H. J. G. (2015). The Effects of a Mobile Phone Application on Quality of Life in Patients With Type 1 Diabetes Mellitus: A Randomized Controlled Trial. *Journal of Diabetes Science and Technology*, *9*(5), 1086–1091. https://doi.org/10.1177/1932296815585871  Ellis, D. A., Carcone, A., I., Slatcher, R., Naar-King, S., Hains, A., Graham, A., & Sibinga, E. (2019). Efficacy of mindfulness-based stress reduction in emerging adults with poorly controlled, type 1 diabetes: A pilot randomized controlled trial. *PEDIATRIC DIABETES*, *20*(2), 226–234. https://doi.org/10.1111/pedi.12807  for the JDRF Canadian Clinical Trial CCTN1102 Study Group, Spaic, T., Mahon, J. L., Hramiak, I., Byers, N., Evans, K., Robinson, T., Lawson, M. L., Malcolm, J., Goldbloom, E. B., & Clarson, C. L. (2013). Multicentre randomized controlled trial of structured transition on diabetes care management compared to standard diabetes care in adolescents and young adults with type 1 diabetes (Transition Trial). *BMC Pediatrics*, *13*(1), 163. https://doi.org/10.1186/1471-2431-13-163  Fountoulakis, S., Papanastasiou, L., Gryparis, A., Markou, A., & Piaditis, G. (2015). Impact and duration effect of telemonitoring on HbA1c, BMI and cost in insulin-treated diabetes mellitus patients with inadequate glycemic control: A randomized controlled study. *Hormones*, *14*(4), 632–643. Scopus. https://doi.org/10.14310/horm.2002.1603  Giannini, C., Lombardo, F., Currò, F., Pomilio, M., Bucciarelli, T., Chiarelli, F., & Mohn, A. (2007). Effects of high-dose vitamin E supplementation on oxidative stress and microalbuminuria in young adult patients with childhood onset type 1 diabetes mellitus. *Diabetes/Metabolism Research and Reviews*, *23*(7), 539–546. Scopus. https://doi.org/10.1002/dmrr.717  Goulet-Gélinas, L., Saade, M.-B., Suppère, C., Fortin, A., Messier, V., Taleb, N., Tagougui, S., Shohoudi, A., Legault, L., Henderson, M., & Rabasa-Lhoret, R. (2021). Comparison of two carbohydrate intake strategies to improve glucose control during exercise in adolescents and adults with type 1 diabetes. *Nutrition, Metabolism, and Cardiovascular Diseases : NMCD*, *31*(4), 1238–1246. https://doi.org/10.1016/j.numecd.2020.12.011  Hermanns, N., Kulzer, B., Ehrmann, D., Bergis-Jurgan, N., & Haak, T. (2013). The effect of a diabetes education programme (PRIMAS) for people with type 1 diabetes: Results of a randomized trial. *Diabetes Research and Clinical Practive*, *102*(3), 149–157. https://doi.org/10.1016/j.diabres.2013.10.009  Howells, L., Wilson, A. C., Skinner, T. C., Newton, R., Morris, A. D., & Greene, S. A. (2002). *Telephone support on glycaemic control in young people with Type 1 diabetes*.  Jansà, M., Vidal, M., Viaplana, J., Levy, I., Conget, I., Gomis, R., & Esmatjes, E. (2006). Telecare in a structured therapeutic education programme addressed to patients with type 1 diabetes and poor metabolic control. *Diabetes Research and Clinical Practice*, *74*(1), 26–32. Scopus. https://doi.org/10.1016/j.diabres.2006.03.005  Kaur, P., Agarwala, A., Makharia, G., Bhatnagar, S., & Tandon, N. (2020). Effect of Gluten-FREE Diet on Metabolic Control and Anthropometric Parameters in Type 1 Diabetes with Subclinical Celiac Disease: A Randomized Controlled Trial. *Endocrine Practice*, *26*(6), 660–667. https://doi.org/10.4158/EP-2019-0479  Mannucci, E., Pala, L., & Rotella, C. M. (2005). Long-term interactive group education for type 1 diabetic patients. *Acta Diabetologica*, *42*(1), 1–6. https://doi.org/10.1007/s00592-005-0167-1  Moattari, M., Hashemi, M., & Dabbaghmanesh, M. H. (2013). The impact of electronic education on metabolic control indicators in patients with diabetes who need insulin: A randomised clinical control trial. *Journal of Clinical Nursing*, *22*(1–2), 32–38. Scopus. https://doi.org/10.1111/j.1365-2702.2012.04200.x  Rudberg, s., Dahlquist, g., Aperia, a., & Persson, b. (1988). reduction of protein-intake decreases glomerular-filtration rate in young type-1 (insulin-dependent) diabetic-patients mainly in hyperfiltering patients. *Diabetologia*, *31*(12), 878–883.  Ruiz de Adana, M. S., Rosa Alhambra-Exposito, M., Munoz-Garach, A., Gonzalez-Molero, I., Colomo, N., Torres-Barea, I., Aguilar-Diosdado, M., Carral, F., Serrano, M., Martinez-Brocca, M. A., Duran, A., Palomares, R., & Diabet Grp SAEDYN Andalusian Soc. (2020). Randomized Study to Evaluate the Impact of Telemedicine Care in Patients With Type 1 Diabetes With Multiple Doses of Insulin and Suboptimal HbA(1c) in Andalusia (Spain): PLATEDIAN Study. *DIABETES CARE*, *43*(2), 337–342. https://doi.org/10.2337/dc19-0739  Santacroce, G., Forlani, G., Giangiulio, S., Galuppi, V., Pagani, M., & Vannini, P. (1990). Long-term effects of eating sucrose on metabolic control of type 1 (insulin-dependent) diabetic outpatients. *Acta Diabetologica Latina*, *27*(4), 365–370. https://doi.org/10.1007/BF02580942  Suh, S., Jean, C., Koo, M., Lee, S. Y., Cho, M. J., Sim, K.-H., Jin, S.-M., Bae, J. C., & Kim, J. H. (2014). A Randomized controlled trial of an internet-based mentoring program for type 1 diabetes patients with inadequate glycemic control. *Diabetes and Metabolism Journal*, *38*(2), 134–142. Scopus. https://doi.org/10.4093/dmj.2014.38.2.134  Vaaler, S., Hanssen, K. F., Dahl-Jørgensen, K., Frølich, W., Aaseth, J., Ødegaard, B., & AagenaE, Ø. (1986). Diabetic Control is Improved by Guar Gum and Wheat Bran Supplementation. *Diabetic Medicine*, *3*(3), 230–233. https://doi.org/10.1111/j.1464-5491.1986.tb00750.x |
| **Reason for exclusion: Another disease** |
| Davis, C. L., Pollock, N. K., Waller, J. L., Allison, J. D., Dennis, B. A., Bassali, R., Melendez, A., Boyle, C. A., & Gower, B. A. (2012). Exercise Dose and Diabetes Risk in Overweight and Obese Children A Randomized Controlled Trial. *JAMA-JOURNAL OF THE AMERICAN MEDICAL ASSOCIATION*, *308*(11), 1103–1112. https://doi.org/10.1001/2012.jama.10762  Eisenberg Colman, M. H., Quick, V. M., Lipsky, L. M., Dempster, K. W., Liu, A., Laffel, L. M. B., Mehta, S. N., & Nansel, T. R. (2018). Disordered Eating Behaviors Are Not Increased by an Intervention to Improve Diet Quality but Are Associated With Poorer Glycemic Control Among Youth With Type 1 Diabetes. *Diabetes Care*, *41*(4), 869–875. https://doi.org/10.2337/dc17-0090  Elbarbary, N. S., Ismail, E. A. R., El-Naggar, A. R., Hamouda, M. H., & El-Hamamsy, M. (2018). The effect of 12 weeks carnosine supplementation on renal functional integrity and oxidative stress in pediatric patients with diabetic nephropathy: A randomized placebo-controlled trial. *PEDIATRIC DIABETES*, *19*(3), 470–477. https://doi.org/10.1111/pedi.12564  Elbarbary, N. S., Ismail, E. A. R., Zaki, M. A., Darwish, Y. W., Ibrahim, M. Z., & El-Hamamsy, M. (2020). Vitamin B complex supplementation as a homocysteine-lowering therapy for early stage diabetic nephropathy in pediatric patients with type 1 diabetes: A randomized controlled trial. *Clinical Nutrition*, *39*(1), 49–56. Scopus. https://doi.org/10.1016/j.clnu.2019.01.006  Ellis, D. A., Naar-King, S., Chen, X., Moltz, K., Cunningham, P. B., & Idalski-Carcone, A. (2012). Multisystemic Therapy Compared to Telephone Support for Youth with Poorly Controlled Diabetes: Findings from a Randomized Controlled Trial. *ANNALS OF BEHAVIORAL MEDICINE*, *44*(2), 207–215. https://doi.org/10.1007/s12160-012-9378-1  Knip, M., Virtanen, S. M., Seppa, K., Ilonen, J., Savilahti, E., Vaarala, O., Reunanen, A., Teramo, K., Hamalainen, A.-M., Paronen, J., Dosch, H.-M., Hakulinen, T., Akerblom, H. K., & Finnish TRIGR Study Grp. (2010). Dietary Intervention in Infancy and Later Signs of Beta-Cell Autoimmunity. N*ew England Journal of Medicine*, *363*(20), 1900–1908. https://doi.org/10.1056/NEJMoa1004809  Kumar, V. S., Wentzell, K. J., Mikkelsen, T., Pentland, A., & Laffel, L. M. (2004). The DAILY (Daily Automated Intensive Log for Youth) Trial: A Wireless, Portable System to Improve Adherence and Glycemic Control in Youth with Diabetes. *Diabetes Technology & Therapeutics*, *6*(4), 445–453. https://doi.org/10.1089/1520915041705893  Petersen, K., Clifton, P., Lister, N., & Keogh, J. (2016). Effect of Improving Dietary Quality on Arterial Stiffness in Subjects with Type 1 and Type 2 Diabetes: A 12 Months Randomised Controlled Trial. *Nutrients*, *8*(6), 382. https://doi.org/10.3390/nu8060382  Shih, E. M., Mittelman, S., Pitukcheewanont, P., Azen, C. G., & Monzavi, R. (2016). Effects of vitamin D repletion on glycemic control and inflammatory cytokines in adolescents with type 1 diabetes: Vitamin D in adolescents with type 1 diabetes. *Pediatric Diabetes*, *17*(1), 36–43. https://doi.org/10.1111/pedi.12238  Sun, S., Puttha, R., Ghezaiel, S., Skae, M., Cooper, C., & Amin, R. (2009). The effect of biopsy-positive silent coeliac disease and treatment with a gluten-free diet on growth and glycaemic control in children with Type 1 diabetes. *Diabetic Medicine*, *26*(12), 1250–1254. Scopus. https://doi.org/10.1111/j.1464-5491.2009.02859.x |
| **Reason for exclusion: Another theme** |
| Anderson, B. J., Wolf, F. M., Burkhart, M. T., Cornell, R. G., & Bacon, G. E. (1989). Effects of peer-group intervention on metabolic control of adolescents with IDDM. Randomized outpatient study. *Diabetes Care*, *12*(3), 179–183. https://doi.org/10.2337/diacare.12.3.179  Bruttomesso, D., Iori, E., Kiwanuka, E., Zanetti, M., Pianta, A., Vettore, M., Tiengo, A., & Tessari, P. (2001). Insulin infusion normalizes fasting and post-prandial albumin and fibrinogen synthesis in Type 1 diabetes mellitus: Original article. *Diabetic Medicine*, *18*(11), 915–920. https://doi.org/10.1046/j.1464-5491.2001.00606.x  Chase, H., Pearson, J., Wightman, C., Roberts, M., Oderberg, A., & Garg, S. (2003). Modem transmission of glucose values reduces the costs and need for clinic visits. *DIABETES CARE*, *26*(5), 1475–1479. https://doi.org/10.2337/diacare.26.5.1475  Fumanelli, J., Franceschi, R., Bonani, M., Orrasch, M., & Cauvin, V. (2020). Treatment of hypoglycemia during prolonged physical activity in adolescents with type 1 diabetes mellitus. *Acta Bio-Medica : Atenei Parmensis*, *91*(4), e2020103. https://doi.org/10.23750/abm.v91i4.8437  Gusso, S., Hofman, P., Lalande, S., Cutfield, W., Robinson, E., & Baldi, J. C. (2008). Impaired stroke volume and aerobic capacity in female adolescents with type 1 and type 2 diabetes mellitus. *Diabetologia*, *51*(7), 1317–1320. https://doi.org/10.1007/s00125-008-1012-1  Jain, S., McVie, R., & Smith, T. (2000). Vitamin E supplementation restores glutathione and malondialdehyde to normal concentrations in erythrocytes of type 1 diabetic children. *Diabetes Care*, *23*(9), 1389–1394. https://doi.org/10.2337/diacare.23.9.1389  Kowalska, A., Piechowiak, K., Ramotowska, A., & Szypowska, A. (2017). Impact of ELKa, the Electronic Device for Prandial Insulin Dose Calculation, on Metabolic Control in Children and Adolescents with Type 1 Diabetes Mellitus: A Randomized Controlled Trial. J*ournal of Diabetes Research*, *2017*. https://doi.org/10.1155/2017/1708148  Maahs, D. M., Chase, H. P., Westfall, E., Slover, R., Huang, S., Shin, J. J., Kaufman, F. R., Pyle, L., & Snell-Bergeon, J. K. (2014). The Effects of Lowering Nighttime and Breakfast Glucose Levels with Sensor-Augmented Pump Therapy on Haemoglobin A1c Levels in Type 1 Diabetes. D*iabetes Technology & Therapeutics*, *16*(5), 284–291. https://doi.org/10.1089/dia.2013.0227  Marrero, D. G., Kronz, K. K., Golden, M. P., Wright, J. C., Orr, D. P., & Fineberg, N. S. (1989). Clinical Evaluation of Computer-Assisted Self-Monitoring of Blood Glucose System. *Diabetes Care*, *12*(5), 345–350. https://doi.org/10.2337/diacare.12.5.345  Newton, K. T., & Ashley, A. (2013). Pilot study of a web-based intervention for adolescents with type 1 diabetes. *Journal of Telemedicine and Telecare*, *19*(8), 443–449. https://doi.org/10.1177/1357633X13512069  Thomas-Dobersen, D. A., Butler-Simon, N., & Fleshner, M. (1993). Evaluation of a weight management intervention program in adolescents with insulin-dependent diabetes mellitus. *Journal of the American Dietetic Association*, *93*(5), 535–540. https://doi.org/10.1016/0002-8223(93)91812-5  Vyas, C., Dalal, L., Talaviya, P., & Saboo, B. (2017). Multiple educational programs improve glycemic control, quality of life with diminishing the impact of diabetes in poorly controlled type 1 diabetics. *Diabetes and Metabolic Syndrome: Clinical Research and Reviews*, *11*, S601–S606. Scopus. https://doi.org/10.1016/j.dsx.2017.04.011 |
| **Reason for exclusion: No data founding** |
| Cakan, N., Ellis, D. A., Templin, T., Frey, M., & Naar-King, S. (2007). The effects of weight status on treatment outcomes in a randomized clinical trial of multisystemic therapy for adolescents with type 1 diabetes and chronically poor metabolic control. *PEDIATRIC DIABETES*, *8*(4), 206–213. https://doi.org/10.1111/j.1399-5448.2007.00273.x  Dluzniak-Golaska, K. (2020). Influence of two different methods of nutrition education on the quality of life in children and adolescents with type 1 diabetes mellitus – a randomized study. *Roczniki Państwowego Zakładu Higieny*, 197–206. https://doi.org/10.32394/rpzh.2020.0117  Fiallo-Scharer, R., Palta, M., Chewning, B. A., Rajamanickam, V., Wysocki, T., Wetterneck, T. B., & Cox, E. D. (2019). Impact of family-centered tailoring of pediatric diabetes self-management resources. *PEDIATRIC DIABETES*, *20*(7), 1016–1024. https://doi.org/10.1111/pedi.12899  Freeman, K. A., Duke, D. C., & Harris, M. A. (2013). Behavioral Health Care for Adolescents with Poorly Controlled Diabetes via Skype: Does Working Alliance Remain Intact? *Journal of Diabetes Science and Technology*, *7*(3), 727–735. https://doi.org/10.1177/193229681300700318  Gilbertson, H. R., Thorburn, A. W., Brand-Miller, J. C., Chondros, P., & Werther, G. A. (2003). Effect of low-glycemic-index dietary advice on dietary quality and food choice in children with type 1 diabetes. *The American Journal of Clinical Nutrition*, *77*(1), 83–90. https://doi.org/10.1093/ajcn/77.1.83  Heyman, E., Toutain, C., Delamarche, P., Berthon, P., Briard, D., Youssef, H., Dekerdanet, M., & Gratas-Delamarche, A. (2007). Exercise training and cardiovascular risk factors in type 1 diabetic adolescent girls. *Pediatric Exercise Science*, *19*(4), 408–419. https://doi.org/10.1123/pes.19.4.408  Kaplan, R. M., Chadwick, M. W., & Schimmel, L. E. (1985). Social Learning Intervention to Promote Metabolic Control in Type I Diabetes Mellitus: Pilot Experiment Results. *Diabetes Care*, *8*(2), 152–155. https://doi.org/10.2337/diacare.8.2.152  Lehmkuhl, H. D., Storch, E. A., Cammarata, C., Meyer, K., Rahman, O., Silverstein, J., Malasanos, T., & Geffken, G. (2010). Telehealth behavior therapy for the management of type 1 diabetes in adolescents. *Journal of Diabetes Science and Technology*, *4*(1), 199–208.  Ljungberg, M., Korpela, R., Ilonen, J., Ludvigsson, J., & Vaarala, O. (2006). Probiotics for the Prevention of Beta Cell Autoimmunity in Children at Genetic Risk of Type 1 Diabetes—The PRODIA Study. *Annals of the New York Academy of Sciences*, *1079*(1), 360–364. https://doi.org/10.1196/annals.1375.055  McGill, D. E., Laffel, L. M., Volkening, L. K., Butler, D. A., Levy, W. L., Wasserman, R. M., & Anderson, B. J. (2020). Text Message Intervention for Teens with Type 1 Diabetes Preserves HbA1c: Results of a Randomized Controlled Trial. D*iabetes Technology & Therapeutics*, *22*(5), 374–382. https://doi.org/10.1089/dia.2019.0350  Mitchell, F., Wilkie, L., Robertson, K., Reilly, J. J., & Kirk, A. (2018). Feasibility and pilot study of an intervention to support active lifestyles in youth with type 1 diabetes: The ActivPals study. *Pediatric Diabetes*, *19*(3), 443–449. https://doi.org/10.1111/pedi.12615  Mulvaney, S. A., Rothman, R. L., Osborn, C. Y., Lybarger, C., Dietrich, M. S., & Wallston, K. A. (2011). Self-management problem solving for adolescents with type 1 diabetes: Intervention processes associated with an Internet program. *Patient Education and Counseling*, *85*(2), 140–142. https://doi.org/10.1016/j.pec.2010.09.018  Nansel, T. R., Iannotti, R. J., Simons-Morton, B. G., Cox, C., Plotnick, L. P., Clark, L. M., & Zeitzoff, L. (2007). Diabetes personal trainer outcomes: Short-term and 1-year outcomes of a diabetes personal trainer intervention among youth with type 1 diabetes. *Diabetes Care*, *30*(10), 2471–2477. https://doi.org/10.2337/dc06-2621  Nansel, T. R., Thomas, D. M., & Liu, A. (2015). Efficacy of a Behavioral Intervention for Pediatric Type 1 Diabetes Across Income. *AMERICAN JOURNAL OF Preventive Medicine*, *49*(6), 930–934. https://doi.org/10.1016/j.amepre.2015.05.006  Nordfeldt, S., Johansson, C., Carlsson, E., & Hammersjö, J.-Å. (2003). Prevention of severe hypoglycaemia in type I diabetes: A randomised controlled population study. *Archives of Disease in Childhood*, *88*(3), 240–245. Scopus. https://doi.org/10.1136/adc.88.3.240  Parthasarathy, L., Khadilkar, V., Chiplonkar, S., & Khadilkar, A. (2019). Effect of Antioxidant Supplementation on Total Antioxidant Status in Indian Children with Type 1 Diabetes. *Journal of Dietary Supplements*, *16*(4), 390–400. Scopus. https://doi.org/10.1080/19390211.2018.1470123  Quirk, H., Glazebrook, C., & Blake, H. (2018). A physical activity intervention for children with type 1 diabetes- steps to active kids with diabetes (STAK-D): A feasibility study. *BMC Pediatrics*, *18*(1), 37. https://doi.org/10.1186/s12887-018-1036-8  Roberts, L., Jones, T. W., & Fournier, P. A. (2002). Exercise training and glycemic control in adolescents with poorly controlled type 1 diabetes mellitus. *Journal of Pediatric Endocrinology & Metabolism : JPEM*, *15*(5), 621–627. https://doi.org/10.1515/jpem.2002.15.5.621  Viklund, G., Örtqvist, E., & Wikblad, K. (2007). Assessment of an empowerment education programme. A randomized study in teenagers with diabetes. *Diabetic Medicine*, *24*(5), 550–556. https://doi.org/10.1111/j.1464-5491.2007.02114.x |
| **Reason for exclusion: No full text found.** |
| Cadario, F., Pozzi, E., Rizzollo, S., Stracuzzi, M., Beux, S., Giorgis, A., Carrera, D., Fullin, F., Riso, S., Rizzo, A. M., Montorfano, G., Bagnati, M., Dianzani, U., Caimmi, P., Bona, G., & Ricordi, C. (2019). Vitamin D and ω-3 supplementations in mediterranean diet during the 1st year of overt type 1 diabetes: A cohort study. *Nutrients*, *11*(9). Scopus. https://doi.org/10.3390/nu11092158  Defrin, R., Josefsberg, Z., & Karp, M. (2004). [The effect of acute physical activity on blood glucose levels of children with insulin-dependent diabetes mellitus]. *Harefuah*, *143*(12), 856–860, 912, 911.  Gupta, S., Sharma, T. K., Kaushik, G. G., & Shekhawat, V. P. S. (2011). Vitamin E Supplementation May Ameliorate Oxidative Stress in Type 1 Diabetes Mellitus Patients. C*linical Laboratory*, *57*(5–6), 379–386.  Matam, P., Kumaraiah, V., Munichoodappa, C., Kumar, K. M., & Aravind, S. (2000). Behavioural intervention in the management of compliance in young type-I diabetics. *The Journal of the Association of Physicians of India*, *48*(10), 967–971.  Ordooei, M., Shojaoddiny-Ardekani, A., Hoseinipoor, S. H., Miroliai, M., & Zare-Zardini, H. (2017). Effect of vitamin D on HbA1c levels of children and adolescents with diabetes mellitus type 1. *Minerva Pediatrica*, *69*(5), 391–395. https://doi.org/10.23736/S0026-4946.16.04145-1  Qadir, K. J., & Zangana, K. O. (2020). Effect of swimming program on glycemic control in male adolescents with type 1 diabetes mellitus. *The Journal of Sports Medicine and Physical Fitness*, *60*(2), 302–307. https://doi.org/10.23736/S0022-4707.19.10053-9  Trento, M., Passera, P., Borgo, E., Tomalino, M., Bajardi, M., Cavallo, F., & Porta, M. (2004). A 5-year randomized controlled study of learning, problem solving ability, and quality of life modifications in people with type 2 diabetes managed by group care. *Diabetes Care*, *27*(3), 670–675. https://doi.org/10.2337/diacare.27.3.670 |
| **Reason for exclusion: Acute effect.** |
| Bally, L., Zueger, T., Buehler, T., Dokumaci, A. S., Speck, C., Pasi, N., Ciller, C., Paganini, D., Feller, K., Loher, H., Rosset, R., Wilhelm, M., Tappy, L., Boesch, C., & Stettler, C. (2016). Metabolic and hormonal response to intermittent high-intensity and continuous moderate intensity exercise in individuals with type 1 diabetes: A randomised crossover study. *Diabetologia*, *59*(4), 776–784. https://doi.org/10.1007/s00125-015-3854-7  Baumer, J. H., Drakeford, J. A., Wadsworth, J., & Savage, D. C. (1982). Effects of dietary fibre and exercise on mid-morning diabetic control—A controlled trial. *Archives of Disease in Childhood*, *57*(12), 905–909. https://doi.org/10.1136/adc.57.12.905  Bozzetto, L., Alderisio, A., Giorgini, M., Barone, F., Giacco, A., Riccardi, G., Rivellese, A. A., & Annuzzi, G. (2016). Extra-Virgin Olive Oil Reduces Glycemic Response to a High-Glycemic Index Meal in Patients With Type 1 Diabetes: A Randomized Controlled Trial. *Diabetes Care*, *39*(4), 518–524. https://doi.org/10.2337/dc15-2189  Farinha, J. B., Ramis, T. R., Vieira, A. F., Macedo, R. C. O., Rodrigues-Krause, J., Boeno, F. P., Schroeder, H. T., Müller, C. H., Boff, W., Krause, M., De Bittencourt, P. I. H. J., & Reischak-Oliveira, A. (2018). Glycemic, inflammatory and oxidative stress responses to different high-intensity training protocols in type 1 diabetes: A randomized clinical trial. *Journal of Diabetes and Its Complications*, *32*(12), 1124–1132. https://doi.org/10.1016/j.jdiacomp.2018.09.008  Faulkner, M. S., Michaliszyn, S. F., & Hepworth, J. T. (2010). A personalized approach to exercise promotion in adolescents with type 1 diabetes. *Pediatric Diabetes*, *11*(3), 166–174. https://doi.org/10.1111/j.1399-5448.2009.00550.x  Horan, P. P., Yarborough, M. C., Besigel, G., & Carlson, D. R. (1990). Computer-assisted self-control of diabetes by adolescents. *The Diabetes Educator*, *16*(3), 205–211. https://doi.org/10.1177/014572179001600311  Kordonouri, O., Hartmann, R., Remus, K., Blaesig, S., Sadeghian, E., & Danne, T. (2012). Benefit of supplementary fat plus protein counting as compared with conventional carbohydrate counting for insulin bolus calculation in children with pump therapy. *Pediatric Diabetes*, *13*(7), 540–544. https://doi.org/10.1111/j.1399-5448.2012.00880.x |
| **Reason for exclusion: Same intervention between groups.** |
| Donaghue, K. C., Pena, M. M., Chan, A. K. F., Blades, B. L., King, J., Storlien, L. H., & Silink, M. (2000). Beneficial effects of increasing monounsaturated fat intake in adolescents with type 1 diabetes. *Diabetes Research and Clinical Practice*, *48*(3), 193–199. https://doi.org/10.1016/S0168-8227(00)00123-6  Franklin, V. L., Waller, A., Pagliari, C., & Greene, S. A. (2006). A randomized controlled trial of Sweet Talk, a text-messaging system to support young people with diabetes. *Diabetic Medicine*, *23*(12), 1332–1338. https://doi.org/10.1111/j.1464-5491.2006.01989.x  Geremia, C., Fornari, A., & Tschiedel, B. (2019). Comparison of the effect of a compact vs a conventional, long-term education program on metabolic control in children and adolescents with type 1 diabetes: A pilot, randomized clinical trial. *Pediatric Diabetes*, *20*(6), 778–784. https://doi.org/10.1111/pedi.12879  Gilbertson, H. R., Brand-Miller, J. C., Thorburn, A. W., Evans, S., Chondros, P., & Werther, G. A. (2001). The effect of flexible low glycemic index dietary advice versus measured carbohydrate exchange diets on glycemic control in children with type 1 diabetes. *Diabetes Care*, *24*(7), 1137–1143. Scopus. https://doi.org/10.2337/diacare.24.7.1137  Harris, M. A., Freeman, K. A., & Duke, D. C. (2015). Seeing Is Believing: Using Skype to Improve Diabetes Outcomes in Youth. *Diabetes Care*, *38*(8), 1427–1434. https://doi.org/10.2337/dc14-2469  Marquard, J., Stahl, A., Lerch, C., Wolters, M., Grotzke-Leweling, M., Mayatepek, E., & Meissner, T. (2011). A prospective clinical pilot-trial comparing the effect of an optimized mixed diet versus a flexible low-glycemic index diet on nutrient intake and HbA(1c) levels in children with type 1 diabetes. J*ournal of Pediatric Endocrinology & Metabolism*, *24*(7–8), 441–447. https://doi.org/10.1515/JPEM.2011.176  Rami, B., Popow, C., Horn, W., Waldhoer, T., & Schober, E. (2006). Telemedical support to improve glycemic control in adolescents with type 1 diabetes mellitus. *European Journal of Pediatrics*, *165*(10), 701–705. https://doi.org/10.1007/s00431-006-0156-6  Wei, C., Allen, R. J., Tallis, P. M., Ryan, F. J., Hunt, L. P., Shield, J. P. H., & Crowne, E. C. (2018). Cognitive behavioural therapy stabilises glycaemic control in adolescents with type 1 diabetes-Outcomes from a randomised control trial. *PEDIATRIC DIABETES*, *19*(1), 106–113. https://doi.org/10.1111/pedi.12519 |
| **Reason for exclusion: Same study sample.** |
| Kahkoska, A. R., Lawson, M. T., Crandell, J., Driscoll, K. A., Kichler, J. C., Seid, M., Maahs, D. M., Kosorok, M. R., & Mayer-Davis, E. J. (2019). Assessment of a Precision Medicine Analysis of a Behavioral Counseling Strategy to Improve Adherence to Diabetes Self-management Among Youth: A Post Hoc Analysis of the FLEX Trial. *JAMA Network Open*, *2*(5), e195137. https://doi.org/10.1001/jamanetworkopen.2019.5137  Lansing, A. H., Stoianova, M., & Stanger, C. (2019). Adolescent Emotional Control Moderates Benefits of a Multicomponent Intervention to Improve Type 1 Diabetes Adherence: A Pilot Randomized Controlled Trial. *Journal of Pediatric Psychology*, *44*(1), 126–136. Scopus. https://doi.org/10.1093/jpepsy/jsy071  Shapiro, J. B., Bryant, F. B., Holmbeck, G. N., Hood, K. K., & Weissberg-Benchell, J. (2021). Do baseline resilience profiles moderate the effects of a resilience-enhancing intervention for adolescents with type I diabetes? *Health Psychology*, *40*(5), 337–346. https://doi.org/10.1037/hea0001076 |

**ESM Table 4.** Characteristics of the included studies.

| **Author, year, country** | **Sample size (girls)** | **Mean age**  **± SD** | **Duration since diagnosis (years)** | **Type of treatment** | **Duration of the intervention (weeks)** | **Measurements (Unit)** |
| --- | --- | --- | --- | --- | --- | --- |
| Campaigne et al., 1984, USA ^1^ | 19 (7) | 8.74 ± 0.52 | 4.50 | AER | 12 | HbA_1c_ (%) |
| Campaigne et al., 1985, USA ^2^ | 14 (8) | 15.64 ± 0.79 | 6.18 | AER | 12 | HbA_1c_ (%) |
| D’Hooge et al., 2011, Belgium ^3^ | 16 (9) | 13.35 ± 2.07 | 5.35 | MC | 20 | HbA_1c_ (%) |
| Gusso et al., 2017, New Zealand ^4^ | 53 (24) | 15.57 ± 1.19 | 6.45 | MC | 20 | HbA_1c_ (%) |
| Hassan et al., 2019, USA ^5^ | 14 (7) | 16.00 ± 1.00 | 6.45 | RT | 12 | HbA_1c_ (%) |
| Huttunen et al., 1989, USA ^6^ | 32 (14) | 12.05 ± 2.55 | 5.15 | AER | 13 | HbA_1c_ (%) |
| Knox et al., 2019, UK ^7^ | 49 (22) | 10.65 ± 1.00 | NR | PA | 24 | HbA_1c_ (%) |
| Landt et al., 1985, USA ^8^ | 15 (8) | 16.02 ± 0.60 | 7.20 | AER | 12 | HbA_1c_ (%) |
| Mohammed et al., 2021, Saudi Arabia ^9^ | 40 (0) | 14.45 ± 1.70 | NR | AER **+** DIET | 12 | HbA_1c_ (%) |
| Nazari et al., 2020, Iran ^10^ | 40 (NR) | 11.11 ± 2.29 | NR | MC | 16 | HbA_1c_ (%) |
| Newton et al., 2009, New Zealand ^11^ | 78 (48) | 14.4 ± 2.37 | NR | PA | 12 | HbA_1c_ (%) |
| Petschnig et al., 2020, Austria ^12^ | 21 (NR) | 11.14 ± 0.75 | 2.72 | RT | 32 | HbA_1c_ (%) |
| Salem et al., 2010, Egypt ^13^ | 196 (121) | 14.70 ± 2.38 | 4.88 | MC | 24 | HbA_1c_ (%) |
| Stratton et al., 1987, USA ^14^ | 16 (8) | 15.30 ± 1.05 | 4.60 | AER | 8 | HbA_1c_ (%) |
| Tomar et al., 2014, Saudi Arabia ^15^ | 24 (0) | 14.27 ± 1.85 | 4.50 | AER | 12 | HbA_1c_ (%) |
| Tunar et al., 2012, Turkey ^16^ | 31 (16) | 14.25 ± 2.02 | 5.65 | RT | 12 | HbA_1c_ (%) |
| Wong et al., 2011, Taiwan ^17^ | 23 (20) | 12.17 ± 1.96 | 3.87 | AER | 12 | HbA_1c_ (%) |
| Abdulrhman et al., 2013, Egypt ^18^ | 20 (10) | 11.35 ± 4.20 | 4.70 | SUP | 12 | HbA_1c_ (%) |
| Agrawal et al., 2011, India ^19^ | 24 (7) | 15.59 ± 8.66 | NR | SUP | 104 | HbA_1c_ (%) |
| Alfonsi et al., 2020, Canada ^20^ | 44 (17) | 13.98 ± 3.17 | 6.26 | CC | 12 | HbA_1c_ (%) |
| Altschuler et al., 2007, Germany ^21^ | 57 (29) | 14.95 ± 1.55 | 6.60 | SUP | 13 | HbA_1c_ (%) |
| Brown et al., 1997, USA ^22^ | 59 (NR) | NR ± NR | NR | NEd | 24 | HbA_1c_ (%) |
| Chatzakis et al., 2019, Greece ^23^ | 80 (41) | 13.50 ± 2.85 | 6.40 | NEd | 20 | HbA_1c_ (%) |
| Donzeau et al., 2020, France ^24^ | 87 (52) | 9.63 ± 3.48 | 4.65 | NEd *vs* CC | 52 | HbA_1c_ (%) |
| Duffus et al., 2022, USA ^25^ | 39 (19) | 15.50 ± 1.71 | 8.35 | DIET | 12 | HbA_1c_ (%) |
| Enander et al., 2012, Sweden ^26^ | 40 (22) | 13.38 ± 3.54 | 8 | CC | 4 | HbA_1c_ (%) |
| Gökşen et al., 2014, Turkey ^27^ | 84 (44) | 16.84 ± 4.85 | 5.55 | CC | 2 | HbA_1c_ (%) |
| Kumar et al., 2021, India ^28^ | 90 (40) | 8.51 ± 4.44 | 0.14 | SUP | 12 | HbA_1c_ (%) |
| Ludvigsson et al., 2001, Sweden ^29^ | 46 (18) | 10.34 ± 3.42 | NR | SUP | 104 | HbA_1c_ (%) |
| Spiegel et al., 2012, USA ^30^ | 66 (25) | 15.10 ± 2.60 | 5.55 | CC | 12 | HbA_1c_ (%) |
| Wang et al., 2022, China ^31^ | 56 (24) | 14.20 ± 4.84 | 6.35 | SUP | 24 | HbA_1c_ (%) |
| Al Ksir et al., 2022, USA ^32^ | 66 (33) | 15.18 ± 1.68 | NR | BT | 24 | HbA_1c_ (%) |
| Ambrosino et al., 2008, USA ^33^ | 87 (52) | 9.91 ± 1.45 | 3.42 | BT | 4 | HbA_1c_ (%) |
| Bakir et al., 2021, Turkey ^34^ | 50 (25) | 14.56 ± 1.85 | 17 | BT | 12 | HbA_1c_ (%) |
| Berndt et al., 2014, Germany ^35^ | 68 (27) | 13.05 ± 2.45 | 5.15 | DEd | 24 | HbA_1c_ (%) |
| Bloomflied et al., 1990, UK ^36^ | 48 (27) | 9.00 ± 3.00 | 2.75 | DEd | 52 | HbA_1c_ (%) |
| Brorsson et al., 2019, Sweden ^37^ | 69 (41) | 14.94 ± 1.83 | NR | BT | 20 | HbA_1c_ (%) |
| Castensøe et al., 2018, Denmark ^38^ | 151 (81) | 17.60 ± 2.65 | NR | DEd | 8 | HbA_1c_ (mmol/mol) |
| Channon et al., 2007, UK ^39^ | 80 (NR) | 15.35 ± 1.07 | 9.15 | BT | 52 | HbA_1c_ (%) |
| Christie et al., 2016, UK ^40^ | 315 (181) | 13.15 ± 2.10 | 5.90 | BT **+** DEd | 16 | HbA_1c_ (%) |
| Edraki et al., 2020, Iran ^41^ | 84 (52) | 14.94 ± 1.83 | 5.25 | BT *vs* DEd | 4 | HbA_1c_ (%) |
| Graue et al., 2005, Norway ^42^ | 101 (47) | 14.41 ± 1.60 | 6.80 | BT | 60 | HbA_1c_ (%) |
| Grey et al., 1998, USA ^43^ | 65 (37) | 15.51 ± 2.20 | 8.10 | BT | 12 | HbA_1c_ (%) |
| Grey et al., 2013, USA ^44^ | 320 (176) | NR ± NR | 6.10 | BT *vs* DEd | 12 | HbA_1c_ (%) |
| Han et al., 2015, USA ^45^ | 30 (17) | 13.70 ± 1.92 | 5.70 | DEd | 12 | HbA_1c_ (%) |
| Hood et al., 2018, USA ^46^ | 264 (158) | 15.74 ± 1.09 | 6.88 | BT *vs* DEd | 18 | HbA_1c_ (%) |
| Husted et al., 2014, Denmark ^47^ | 71 (43) | 14.76 ± 1.40 | 5.70 | BT | 86 | HbA_1c_ (%) |
| Ibrahim et al., 2021, France ^48^ | 92 (57) | 14.95 ± 2.06 | 7.75 | DEd | 24 | HbA_1c_ (%) |
| Izquierdo et al., 2009, USA ^49^ | 41 (NR) | 10.10 ± 2.32 | 4.90 | DEd | 24 | HbA_1c_ (%) |
| Jaser et al., 2019, USA ^50^ | 120 (63) | 14.83 ± 1.45 | 5.85 | BT *vs* DEd | 8 | HbA_1c_ (%) |
| Jaser et al., 2020, USA ^51^ | 39 (20) | 9.18 ± 1.82 | 6.94 | SLEd | 4 | HbA_1c_ (%) |
| Katz et al., 2014, USA ^52^ | 153 (85) | 12.59 ± 2.25 | 6.18 | DEd *vs* BT | 52 | HbA_1c_ (%) |
| Kaushal et al., 2020, USA ^53^ | 165 (78) | 15.65 ± 2.10 | 7.10 | DEd | 25 | HbA_1c_ (%) |
| Klee et al., 2018, Switzerland ^54^ | 55 (24) | 13.65 ± 2.40 | 6.50 | DEd | 12 | HbA_1c_ (%) |
| Lawson et al., 2005, Canada ^55^ | 46 (19) | 15.20 ± 1.25 | 6.50 | DEd | 24 | HbA_1c_ (%) |
| Maranda et al., 2015, USA ^56^ | 28 (12) | 14.17 ± 1.96 | 5.85 | BT | 12 | HbA_1c_ (%) |
| Massouh et al., 1989, USA ^57^ | 33 (18) | 12.84 ± 0.84 | 4.30 | BT **+** DEd | 2 | HbA_1c_ (%) |
| Mayer-Davis et al., 2018, USA ^58^ | 258 (99) | 14.85 ± 1.10 | 6.45 | BT | 72 | HbA_1c_ (mmol/mol) |
| Murphy et al., 2012, Australia ^59^ | 305 (159) | 13.15 ± 1.95 | 5.60 | BT **+** DEd **+** FT | 24 | HbA_1c_ (%) |
| Najmi et al., 2013, Iran ^60^ | 113 (50) | 14.74 ± 1.80 | NR | BT | 8 | HbA_1c_ (%) |
| Nunn et al., 2006, Australia ^61^ | 123 (64) | 11.90 ± 3.34 | 3.65 | DEd | 28 | HbA_1c_ (%) |
| Panagiotopoulos et al., 2003, Canada ^62^ | 50 (30) | 14.10 ± 1.60 | 6 | DEd | 24 | HbA_1c_ (%) |
| Price et al., 2016, UK ^63^ | 396 (239) | 13.81 ± 1.53 | 5.74 | NEd + BT | 1 | HbA_1c_ (%) |
| Robling et al., 2012, UK ^64^ | 689 (347) | 10.54 ± 2.80 | 5.10 | BT | 52 | HbA_1c_ (%) |
| Saßmann et al., 2012, Germany ^65^ | 65 (NR) | 6.14 ± 2.13 | 2.60 | FT | 6 | HbA_1c_ (%) |
| Schache et al., 2020, New Zealand ^66^ | 80 (32) | 12.15 ± 1.80 | NR | BT | 8 | HbA_1c_ (%) |
| Schiaffini et al., 2015, Italy ^67^ | 29 (18) | 12.99 ± 0.85 | 4.35 | DEd **+** DIET **+** AER *vs* DIET **+** AER | 104 | HbA_1c_ (%) |
| Serlachius et al., 2016, Australia ^68^ | 147 (79) | 14.33 ± 1.10 | 5.88 | BT | 5 | HbA_1c_ (%) |
| Stanger et al., 2018, USA ^69^ | 61 (26) | 15.05 ± 1.45 | 6.20 | DEd | 25 | HbA_1c_ (%) |
| Tuomaala et al., 2021, Finland ^70^ | 47 (21) | 14.60 ± 0.85 | 8.05 | BT | 52 | HbA_1c_ (mmol/mol) |
| Wang et al., 2010, USA ^71^ | 44 (22) | 15.46 ± 1.56 | 7.15 | BT *vs* DEd | 24 | HbA_1c_ (%) |
| Whittemore et al., 2016, USA ^72^ | 124 (62) | 12.15 ± 1.10 | 4.84 | BT *vs* DEd | 4 | HbA_1c_ (%) |
| Wysocki et al., 2006, USA ^73^ | 104 (47) | 14.04 ± 1.90 | 5.60 | BT *vs* DEd | 52 | HbA_1c_ (%) |
| Zarifsaniey et al., 2022, Iran ^74^ | 66 (41) | 12.54 ± 3.44 | 8.00 | DEd | 12 | HbA_1c_ (%) |
| *Note*: **AER**, Aerobic exercise training; **BT,** Behavioural therapy; **CC,** Carbohydrate counting; **DEd**, Diabetes Education; **DIET**, Diet; **FT**, Family therapy; **MC**, Multicomponent exercise training; **NEd**, Nutritional education; **NR,** Not reported; **PA**, Physical activity promotion; **RT**, Resistance exercise training; **SD**, standard deviation; **SLEd**, Sleep education; **SUP**, Nutritional supplements. | | | | | | |

**ESM Method 1. Characteristics of the included studies.**

Of the 74 randomised controlled trials (RCT), 11 compared two interventions ^5,24,40,41,44,46,50,67,71,75,76^, one had three arms ^73^, and two had four arms ^9,60^. Most included both sexes, but eight only enrolled boys ^9,10,12,22,49,65,77,78^.

Interventions encompassed aerobic exercise, resistance training, multicomponent exercise, physical activity promotion, diet, supplements, nutrition education, carbohydrate counting, behavioural therapy, diabetes education, sleep education, family therapy, and combinations thereof ^79^. Duration ranged from four ^33,41,51,76^ to 104 weeks ^29,80^, with frequency of 1-7 times/week and session length of 20-90 minutes supervised or unsupervised.

Glycated haemoglobin (HbA1c) was measured via specialized laboratories or biochemistry kits. Insulin data was collected from pumps or administration records.

**ESM Table 5:** League table of the network meta-analysis for daily insulin dose requirement.


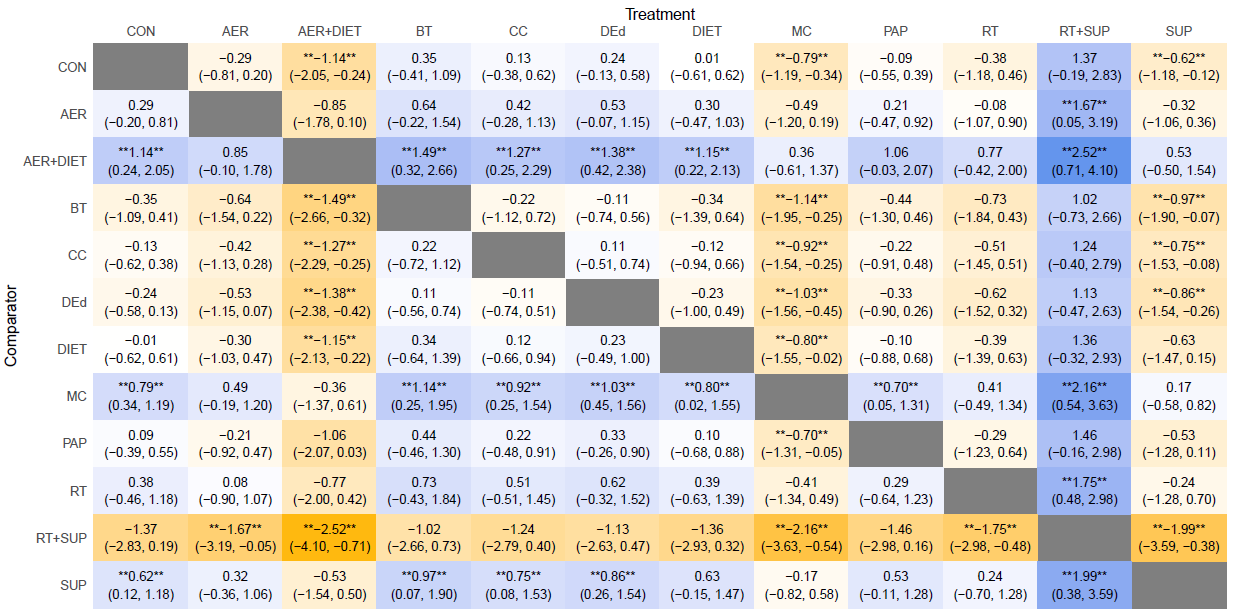


*Note*: Values correspond to standardised mean difference and 95% credible interval between column and row. The table is read from top to bottom and from left to right. Negative values ​​indicate a favourable reduction in daily insulin dose requirement towards the first intervention of each comparison. (e.g., multicomponent training had a reduction in daily insulin dose requirement of 0.79 compared to the control group). **AER**, Aerobic; **BT**, Behavioural Therapy; **CC**, Carbohydrate Counting; **CON**, Control; **DEd**, Diabetes Education; **MC**, Multicomponent; **PA**, Physical Activity; **RT**, Resistance Training; **SUP**, Nutritional Supplements. ** *p*-value < 0.05.

**ESM Table 6:** Complementary league table from the network meta-analysis for changes in % glycated haemoglobin.


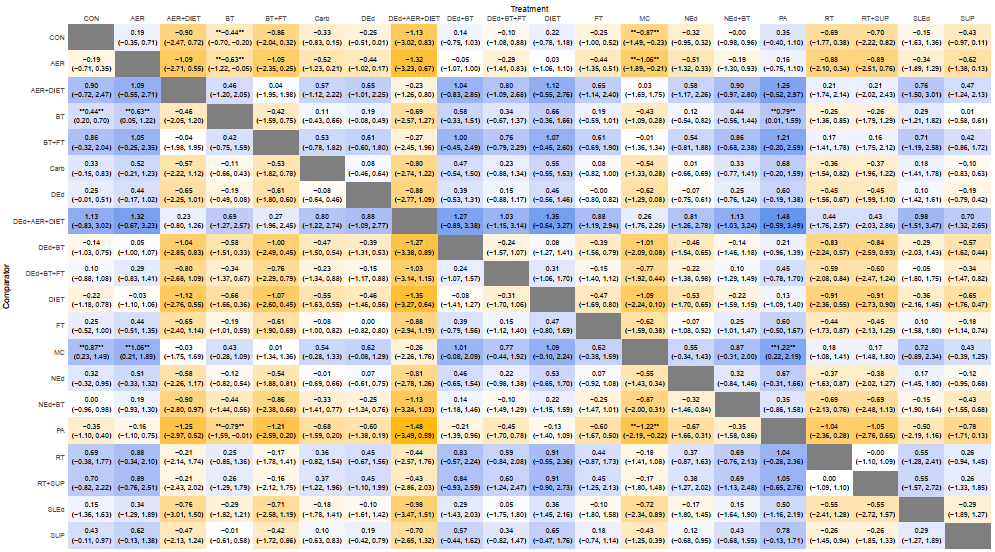


*Note*: The values ​​correspond to the changes in the percentage (%) of HbA1c, after adjusting all units of measurement, and to the 95% credibility interval between column and row. The table is read from top to bottom and from left to right. Negative values ​​indicate a favourable reduction in glycated haemoglobin (HbA1c) towards the first intervention of each comparison. (e.g., multicomponent training had an HbA_1c_ reduction of 0.87 compared to the control group). **AER**, Aerobic; **BT**, Behavioural Therapy; **Carb**, Carbohydrate Counting; **CON**, Control; **DEd**, Diabetes Education; **FT**, Family Therapy; **MC**, Multicomponent; **NEd,** Nutritional Education; **PA**, Physical Activity; **RT**, Resistance Training; **SLEd,** Sleep Education; **SUP,** Nutritional Supplements. ***p*-value < 0.05.

**ESM Table 7.** Meta-regression analyses by participants’ age, number of girls, intervention duration, and disease duration, for all interventions (only versus control) included in the network meta-analysis.

|  | **Nonpharmacological interventions versus control** | | | |
| --- | --- | --- | --- | --- |
| Predictor | *k* | *B* | 95% lower CrI | 95% upper CrI |
| Participants’ age (years) | 67 | −0.019 | −0.027 | −0.011 |
| Girls (number) | 61 | −0.001 | −0.003 | 0.0003 |
| Intervention duration (weeks) | 69 | −0.007 | −0.010 | −0.004 |
| Disease duration (years) | 54 | −0.038 | −0.056 | −0.020 |

*Note*: ***B****,* Unstandardised beta coefficient; **CrI**, credible interval. ***k***, number of studies.

**ESM Table 8.** Meta-regression analyses by participants’ age, number of girls, intervention duration, and disease duration, for those intervention types with 10 or more studies.

|  | **Behavioural therapy** | | | |
| --- | --- | --- | --- | --- |
| Predictor | *k* | *B* | 95% lower CrI | 95% upper CrI |
| Participants’ age (years) | 15 | −0.025 | −0.040 | −0.011 |
| Girls (number) | 14 | −0.001 | −0.004 | 0.002 |
| Intervention duration (weeks) | 15 | −0.008 | −0.012 | −0.002 |
| Disease duration (years) | 12 | −0.013 | −0.085 | −0.025 |
|  | **Diabetes education** | | | |
| Predictor | *k* | *B* | 95% lower CrI | 95% upper CrI |
| Participants’ age (years) | 13 | −0.007 | −0.023 | 0.008 |
| Girls (number) | 12 | −0.001 | −0.005 | 0.004 |
| Intervention duration (weeks) | 13 | −0.008 | −0.016 | 0.0002 |
| Disease duration (years) | 13 | −0.013 | −0.047 | 0.021 |

*Note*: ***B****,* Unstandardised beta coefficient**; CrI**, credible interval; ***k***, number of studies.

**ESM Table 9.** Confidence in Network Meta-Analysis (CINeMA) confidence rating.

| **Comparison** | ***k*** | **Within-study bias** | **Reporting bias** | **Indirectness** | **Imprecision** | **Heterogeneity** | **Incoherence** | **Confidence Rating** |
| --- | --- | --- | --- | --- | --- | --- | --- | --- |
| AER:AER+DIET | 1 | Some concerns | Some concerns | No concerns | Some concerns | No concerns | No concerns | Low |
| AER:CON | 8 | Some concerns | Some concerns | No concerns | No concerns | No concerns | No concerns | Moderate |
| AER:DIET | 1 | Some concerns | Some concerns | No concerns | Some concerns | No concerns | No concerns | Low |
| AER+DIET:CON | 1 | Some concerns | Some concerns | No concerns | Some concerns | No concerns | No concerns | Low |
| AER+DIET:DEd+AER+DIET | 1 | Some concerns | Some concerns | No concerns | Some concerns | No concerns | No concerns | Low |
| AER+DIET:DIET | 1 | Some concerns | Some concerns | No concerns | Some concerns | No concerns | No concerns | Low |
| BT:BT+FT | 1 | Some concerns | Some concerns | No concerns | No concerns | Some concerns | No concerns | Low |
| BT:CON | 16 | Some concerns | Some concerns | No concerns | No concerns | Some concerns | No concerns | Low |
| BT:DEd | 8 | Some concerns | Some concerns | No concerns | No concerns | No concerns | No concerns | Moderate |
| BT:FT | 1 | Some concerns | Some concerns | No concerns | No concerns | No concerns | No concerns | Moderate |
| BT+FT:CON | 1 | Some concerns | Some concerns | No concerns | Some concerns | No concerns | No concerns | Low |
| BT+FT:FT | 1 | Some concerns | Some concerns | No concerns | Some concerns | No concerns | No concerns | Low |
| CC:CON | 4 | Some concerns | Some concerns | No concerns | No concerns | Some concerns | No concerns | Low |
| CON:DEd | 14 | Some concerns | High risk | No concerns | No concerns | No concerns | No concerns | Low |
| CON:DEd+BT | 2 | Major concerns | Some concerns | No concerns | No concerns | No concerns | No concerns | Low |
| CON:DEd+BT+FT | 1 | Some concerns | Some concerns | No concerns | No concerns | Some concerns | No concerns | Low |
| CON:DIET | 2 | Some concerns | Some concerns | No concerns | No concerns | Some concerns | No concerns | Low |
| CON:FT | 2 | Some concerns | Some concerns | No concerns | No concerns | Some concerns | No concerns | Low |
| CON:MC | 4 | Major concerns | Some concerns | No concerns | No concerns | Some concerns | No concerns | Very low |
| CON:NEd | 2 | Major concerns | Some concerns | No concerns | No concerns | Some concerns | No concerns | Very low |
| CON:NEd+BT | 1 | Major concerns | Some concerns | No concerns | No concerns | No concerns | No concerns | Low |
| CON:PA | 2 | Some concerns | Some concerns | No concerns | Some concerns | No concerns | No concerns | Low |
| CON:RT | 2 | Some concerns | Some concerns | No concerns | Some concerns | No concerns | No concerns | Low |
| CON:SLEd | 1 | Some concerns | Some concerns | No concerns | Some concerns | Some concerns | No concerns | Very low |
| CON:SUP | 6 | Some concerns | Some concerns | No concerns | No concerns | Some concerns | No concerns | Low |
| CC:NEd | 1 | Major concerns | Some concerns | No concerns | No concerns | Some concerns | No concerns | Very low |
| RT:RT+SUP | 1 | Some concerns | Some concerns | No concerns | Major concerns | No concerns | No concerns | Very low |
| AER:BT | 0 | Some concerns | Some concerns | No concerns | No concerns | Some concerns | No concerns | Low |
| AER:BT+FT | 0 | Some concerns | Some concerns | No concerns | Some concerns | No concerns | No concerns | Low |
| AER:CC | 0 | Some concerns | Some concerns | No concerns | Some concerns | No concerns | No concerns | Low |
| AER:DEd | 0 | Some concerns | Some concerns | No concerns | No concerns | No concerns | No concerns | Moderate |
| AER:DEd+AER+DIET | 0 | Some concerns | Some concerns | No concerns | Some concerns | No concerns | No concerns | Low |
| AER:DEd+BT | 0 | Major concerns | Some concerns | No concerns | No concerns | Some concerns | No concerns | Very low |
| AER:DEd+BT+FT | 0 | Some concerns | Some concerns | No concerns | No concerns | Major concerns | No concerns | Very low |
| AER:FT | 0 | Some concerns | Some concerns | No concerns | No concerns | Some concerns | No concerns | Low |
| AER:MC | 0 | Some concerns | Some concerns | No concerns | Some concerns | No concerns | No concerns | Low |
| AER:NEd | 0 | Some concerns | Some concerns | No concerns | No concerns | Some concerns | No concerns | Low |
| AER:NEd+BT | 0 | Major concerns | Some concerns | No concerns | No concerns | Some concerns | No concerns | Very low |
| AER:PA | 0 | Some concerns | Some concerns | No concerns | Some concerns | No concerns | No concerns | Low |
| AER:RT | 0 | Some concerns | Some concerns | No concerns | Some concerns | No concerns | No concerns | Low |
| AER:RT+SUP | 0 | Some concerns | Some concerns | No concerns | Major concerns | No concerns | No concerns | Very low |
| AER:SLEd | 0 | Some concerns | Some concerns | No concerns | Major concerns | No concerns | No concerns | Very low |
| AER:SUP | 0 | Some concerns | Some concerns | No concerns | No concerns | Some concerns | No concerns | Low |
| AER+DIET:BT | 0 | Some concerns | Some concerns | No concerns | Some concerns | Some concerns | No concerns | Very low |
| AER+DIET:BT+FT | 0 | Some concerns | Some concerns | No concerns | Major concerns | No concerns | No concerns | Very low |
| AER+DIET:CC | 0 | Some concerns | Some concerns | No concerns | Major concerns | No concerns | No concerns | Very low |
| AER+DIET:DEd | 0 | Some concerns | Some concerns | No concerns | Some concerns | No concerns | No concerns | Low |
| AER+DIET:DEd+BT | 0 | Some concerns | Some concerns | No concerns | Some concerns | No concerns | No concerns | Low |
| AER+DIET:DEd+BT+FT | 0 | Some concerns | Some concerns | No concerns | Some concerns | No concerns | No concerns | Low |
| AER+DIET:FT | 0 | Some concerns | Some concerns | No concerns | Some concerns | Some concerns | No concerns | Very low |
| AER+DIET:MC | 0 | Some concerns | Some concerns | No concerns | Major concerns | No concerns | No concerns | Very low |
| AER+DIET:NEd | 0 | Some concerns | Some concerns | No concerns | Some concerns | Some concerns | No concerns | Very low |
| AER+DIET:NEd+BT | 0 | Some concerns | Some concerns | No concerns | Some concerns | No concerns | No concerns | Low |
| AER+DIET:PA | 0 | Some concerns | Some concerns | No concerns | Some concerns | No concerns | No concerns | Low |
| AER+DIET:RT | 0 | Some concerns | Some concerns | No concerns | Major concerns | No concerns | No concerns | Very low |
| AER+DIET:RT+SUP | 0 | Some concerns | Some concerns | No concerns | Major concerns | No concerns | No concerns | Very low |
| AER+DIET:SLEd | 0 | Some concerns | Some concerns | No concerns | Some concerns | Some concerns | No concerns | Very low |
| AER+DIET:SUP | 0 | Some concerns | Some concerns | No concerns | Some concerns | Some concerns | No concerns | Very low |
| BT:CC | 0 | Some concerns | Some concerns | No concerns | No concerns | No concerns | No concerns | Moderate |
| BT:DEd+AER+DIET | 0 | Some concerns | Some concerns | No concerns | Some concerns | No concerns | No concerns | Low |
| BT:DEd+BT | 0 | Some concerns | Some concerns | No concerns | Some concerns | No concerns | No concerns | Low |
| BT:DEd+BT+FT | 0 | Some concerns | Some concerns | No concerns | Some concerns | No concerns | No concerns | Low |
| BT:DIET | 0 | Some concerns | Some concerns | No concerns | Some concerns | No concerns | No concerns | Low |
| BT:MC | 0 | Some concerns | Some concerns | No concerns | No concerns | Some concerns | No concerns | Low |
| BT:NEd | 0 | Some concerns | Some concerns | No concerns | No concerns | Some concerns | No concerns | Low |
| BT:NEd+BT | 0 | Some concerns | Some concerns | No concerns | Some concerns | No concerns | No concerns | Low |
| BT:PA | 0 | Some concerns | Some concerns | No concerns | No concerns | Some concerns | No concerns | Low |
| BT:RT | 0 | Some concerns | Some concerns | No concerns | No concerns | Major concerns | No concerns | Very low |
| BT:RT+SUP | 0 | Some concerns | Some concerns | No concerns | Major concerns | No concerns | No concerns | Very low |
| BT:SLEd | 0 | Some concerns | Some concerns | No concerns | Some concerns | No concerns | No concerns | Low |
| BT:SUP | 0 | Some concerns | Some concerns | No concerns | No concerns | No concerns | No concerns | Moderate |
| BT+FT:CC | 0 | Some concerns | Some concerns | No concerns | No concerns | Major concerns | No concerns | Very low |
| BT+FT:DEd | 0 | Some concerns | Some concerns | No concerns | Some concerns | No concerns | No concerns | Low |
| BT+FT:DEd+AER+DIET | 0 | Some concerns | Some concerns | No concerns | Major concerns | No concerns | No concerns | Very low |
| BT+FT:DEd+BT | 0 | Some concerns | Some concerns | No concerns | Some concerns | No concerns | No concerns | Low |
| BT+FT:DEd+BT+FT | 0 | Some concerns | Some concerns | No concerns | Some concerns | No concerns | No concerns | Low |
| BT+FT:DIET | 0 | Some concerns | Some concerns | No concerns | Some concerns | No concerns | No concerns | Low |
| BT+FT:MC | 0 | Some concerns | Some concerns | No concerns | No concerns | Major concerns | No concerns | Very low |
| BT+FT:NEd | 0 | Some concerns | Some concerns | No concerns | Some concerns | No concerns | No concerns | Low |
| BT+FT:NEd+BT | 0 | Some concerns | Some concerns | No concerns | Some concerns | No concerns | No concerns | Low |
| BT+FT:PA | 0 | Some concerns | Some concerns | No concerns | Some concerns | No concerns | No concerns | Low |
| BT+FT:RT | 0 | Some concerns | Some concerns | No concerns | Some concerns | Some concerns | No concerns | Very low |
| BT+FT:RT+SUP | 0 | Some concerns | Some concerns | No concerns | Major concerns | No concerns | No concerns | Very low |
| BT+FT:SLEd | 0 | Some concerns | Some concerns | No concerns | Some concerns | No concerns | No concerns | Low |
| BT+FT:SUP | 0 | Some concerns | Some concerns | No concerns | Some concerns | No concerns | No concerns | Low |
| CON:DEd+AER+DIET | 0 | Some concerns | Some concerns | No concerns | Some concerns | No concerns | No concerns | Low |
| CON:RT+SUP | 0 | Some concerns | Some concerns | No concerns | Major concerns | No concerns | No concerns | Very low |
| CC:DEd | 0 | Some concerns | Some concerns | No concerns | No concerns | Some concerns | No concerns | Low |
| CC:DEd+AER+DIET | 0 | Some concerns | Some concerns | No concerns | Some concerns | Some concerns | No concerns | Very low |
| CC:DEd+BT | 0 | Major concerns | Some concerns | No concerns | Some concerns | No concerns | No concerns | Very low |
| CC:DEd+BT+FT | 0 | Some concerns | Some concerns | No concerns | Some concerns | No concerns | No concerns | Low |
| CC:DIET | 0 | Some concerns | Some concerns | No concerns | Some concerns | No concerns | No concerns | Low |
| CC:FT | 0 | Some concerns | Some concerns | No concerns | No concerns | Some concerns | No concerns | Low |
| CC:MC | 0 | Major concerns | Some concerns | No concerns | No concerns | Some concerns | No concerns | Very low |
| CC:NEd+BT | 0 | Major concerns | Some concerns | No concerns | Some concerns | No concerns | No concerns | Very low |
| CC:PA | 0 | Some concerns | Some concerns | No concerns | No concerns | Some concerns | No concerns | Low |
| CC:RT | 0 | Some concerns | Some concerns | No concerns | Some concerns | Some concerns | No concerns | Very low |
| CC:RT+SUP | 0 | Some concerns | Some concerns | No concerns | Major concerns | No concerns | No concerns | Very low |
| CC:SLEd | 0 | Some concerns | Some concerns | No concerns | Some concerns | No concerns | No concerns | Low |
| CC:SUP | 0 | Some concerns | Some concerns | No concerns | No concerns | No concerns | No concerns | Moderate |
| DEd:DEd+AER+DIET | 0 | Some concerns | Some concerns | No concerns | Some concerns | No concerns | No concerns | Low |
| DEd:DEd+BT | 0 | Major concerns | Some concerns | No concerns | No concerns | Some concerns | No concerns | Very low |
| DEd:DEd+BT+FT | 0 | Some concerns | Some concerns | No concerns | No concerns | Some concerns | No concerns | Low |
| DEd:DIET | 0 | Some concerns | Some concerns | No concerns | Some concerns | No concerns | No concerns | Low |
| DEd:FT | 0 | Some concerns | Some concerns | No concerns | No concerns | Some concerns | No concerns | Low |
| DEd:MC | 0 | Some concerns | Some concerns | No concerns | No concerns | Some concerns | No concerns | Low |
| DEd:NEd | 0 | Some concerns | Some concerns | No concerns | No concerns | No concerns | No concerns | Moderate |
| DEd:NEd+BT | 0 | Major concerns | Some concerns | No concerns | No concerns | Some concerns | No concerns | Very low |
| DEd:PA | 0 | Some concerns | Some concerns | No concerns | Some concerns | No concerns | No concerns | Low |
| DEd:RT | 0 | Some concerns | Some concerns | No concerns | Some concerns | No concerns | No concerns | Low |
| DEd:RT+SUP | 0 | Some concerns | Some concerns | No concerns | Major concerns | No concerns | No concerns | Very low |
| DEd:SLEd | 0 | Some concerns | Some concerns | No concerns | Some concerns | Some concerns | No concerns | Very low |
| DEd:SUP | 0 | Some concerns | Some concerns | No concerns | No concerns | No concerns | No concerns | Moderate |
| DEd+AER+DIET:DEd+BT | 0 | Some concerns | Some concerns | No concerns | Some concerns | No concerns | No concerns | Low |
| DEd+AER+DIET:DEd+BT+FT | 0 | Some concerns | Some concerns | No concerns | Some concerns | No concerns | No concerns | Low |
| DEd+AER+DIET:DIET | 0 | Some concerns | Some concerns | No concerns | Some concerns | No concerns | No concerns | Low |
| DEd+AER+DIET:FT | 0 | Some concerns | Some concerns | No concerns | Some concerns | No concerns | No concerns | Low |
| DEd+AER+DIET:MC | 0 | Some concerns | Some concerns | No concerns | Major concerns | No concerns | No concerns | Very low |
| DEd+AER+DIET:NEd | 0 | Some concerns | Some concerns | No concerns | Some concerns | No concerns | No concerns | Low |
| DEd+AER+DIET:NEd+BT | 0 | Some concerns | Some concerns | No concerns | Some concerns | No concerns | No concerns | Low |
| DEd+AER+DIET:PA | 0 | Some concerns | Some concerns | No concerns | Some concerns | No concerns | No concerns | Low |
| DEd+AER+DIET:RT | 0 | Some concerns | Some concerns | No concerns | Some concerns | Some concerns | No concerns | Very low |
| DEd+AER+DIET:RT+SUP | 0 | Some concerns | Some concerns | No concerns | Major concerns | No concerns | No concerns | Very low |
| DEd+AER+DIET:SLEd | 0 | Some concerns | Some concerns | No concerns | Some concerns | No concerns | No concerns | Low |
| DEd+AER+DIET:SUP | 0 | Some concerns | Some concerns | No concerns | Some concerns | No concerns | No concerns | Low |
| DEd+BT:DEd+BT+FT | 0 | Major concerns | Some concerns | No concerns | Some concerns | No concerns | No concerns | Very low |
| DEd+BT:DIET | 0 | Some concerns | Some concerns | No concerns | Some concerns | Some concerns | No concerns | Very low |
| DEd+BT:FT | 0 | Major concerns | Some concerns | No concerns | Some concerns | No concerns | No concerns | Very low |
| DEd+BT:MC | 0 | Major concerns | Some concerns | No concerns | Some concerns | No concerns | No concerns | Very low |
| DEd+BT:NEd | 0 | Major concerns | Some concerns | No concerns | Some concerns | No concerns | No concerns | Very low |
| DEd+BT:NEd+BT | 0 | Major concerns | Some concerns | No concerns | No concerns | Major concerns | No concerns | Very low |
| DEd+BT:PA | 0 | Some concerns | Some concerns | No concerns | Some concerns | No concerns | No concerns | Low |
| DEd+BT:RT | 0 | Major concerns | Some concerns | No concerns | Some concerns | No concerns | No concerns | Very low |
| DEd+BT:RT+SUP | 0 | Some concerns | Some concerns | No concerns | Major concerns | No concerns | No concerns | Very low |
| DEd+BT:SLEd | 0 | Major concerns | Some concerns | No concerns | Some concerns | Some concerns | No concerns | Very low |
| DEd+BT:SUP | 0 | Major concerns | Some concerns | No concerns | Some concerns | No concerns | No concerns | Very low |
| DEd+BT+FT:DIET | 0 | Some concerns | Some concerns | No concerns | Some concerns | Some concerns | No concerns | Very low |
| DEd+BT+FT:FT | 0 | Some concerns | Some concerns | No concerns | Some concerns | No concerns | No concerns | Low |
| DEd+BT+FT:MC | 0 | Some concerns | Some concerns | No concerns | Some concerns | No concerns | No concerns | Low |
| DEd+BT+FT:NEd | 0 | Some concerns | Some concerns | No concerns | Some concerns | No concerns | No concerns | Low |
| DEd+BT+FT:NEd+BT | 0 | Major concerns | Some concerns | No concerns | Some concerns | Some concerns | No concerns | Very low |
| DEd+BT+FT:PA | 0 | Some concerns | Some concerns | No concerns | Some concerns | No concerns | No concerns | Low |
| DEd+BT+FT:RT | 0 | Some concerns | Some concerns | No concerns | Some concerns | No concerns | No concerns | Low |
| DEd+BT+FT:RT+SUP | 0 | Some concerns | Some concerns | No concerns | Major concerns | No concerns | No concerns | Very low |
| DEd+BT+FT:SLEd | 0 | Some concerns | Some concerns | No concerns | Major concerns | No concerns | No concerns | Very low |
| DEd+BT+FT:SUP | 0 | Some concerns | Some concerns | No concerns | Some concerns | No concerns | No concerns | Low |
| DIET:FT | 0 | Some concerns | Some concerns | No concerns | Some concerns | No concerns | No concerns | Low |
| DIET:MC | 0 | Some concerns | Some concerns | No concerns | Some concerns | No concerns | No concerns | Low |
| DIET:NEd | 0 | Some concerns | Some concerns | No concerns | Some concerns | No concerns | No concerns | Low |
| DIET:NEd+BT | 0 | Some concerns | Some concerns | No concerns | Some concerns | Some concerns | No concerns | Very low |
| DIET:PA | 0 | Some concerns | Some concerns | No concerns | Some concerns | No concerns | No concerns | Low |
| DIET:RT | 0 | Some concerns | Some concerns | No concerns | Some concerns | No concerns | No concerns | Low |
| DIET:RT+SUP | 0 | Some concerns | Some concerns | No concerns | Major concerns | No concerns | No concerns | Very low |
| DIET:SLEd | 0 | Some concerns | Some concerns | No concerns | Major concerns | No concerns | No concerns | Very low |
| DIET:SUP | 0 | Some concerns | Some concerns | No concerns | Some concerns | No concerns | No concerns | Low |
| FT:MC | 0 | Major concerns | Some concerns | No concerns | Some concerns | No concerns | No concerns | Very low |
| FT:NEd | 0 | Some concerns | Some concerns | No concerns | No concerns | Some concerns | No concerns | Low |
| FT:NEd+BT | 0 | Major concerns | Some concerns | No concerns | Some concerns | No concerns | No concerns | Very low |
| FT:PA | 0 | Some concerns | Some concerns | No concerns | Some concerns | No concerns | No concerns | Low |
| FT:RT | 0 | Some concerns | Some concerns | No concerns | Major concerns | No concerns | No concerns | Very low |
| FT:RT+SUP | 0 | Some concerns | Some concerns | No concerns | Major concerns | No concerns | No concerns | Very low |
| FT:SLEd | 0 | Some concerns | Some concerns | No concerns | Some concerns | Some concerns | No concerns | Very low |
| FT:SUP | 0 | Some concerns | Some concerns | No concerns | No concerns | Some concerns | No concerns | Low |
| MC:NEd | 0 | Major concerns | Some concerns | No concerns | Some concerns | No concerns | No concerns | Very low |
| MC:NEd+BT | 0 | Major concerns | Some concerns | No concerns | Some concerns | No concerns | No concerns | Very low |
| MC:PA | 0 | Some concerns | Some concerns | No concerns | No concerns | Some concerns | No concerns | Low |
| MC:RT | 0 | Some concerns | Some concerns | No concerns | Some concerns | No concerns | No concerns | Low |
| MC:RT+SUP | 0 | Some concerns | Some concerns | No concerns | Major concerns | No concerns | No concerns | Very low |
| MC:SLEd | 0 | Some concerns | Some concerns | No concerns | Some concerns | No concerns | No concerns | Low |
| MC:SUP | 0 | Major concerns | Some concerns | No concerns | No concerns | Some concerns | No concerns | Very low |
| NEd:NEd+BT | 0 | Major concerns | Some concerns | No concerns | Some concerns | No concerns | No concerns | Very low |
| NEd:PA | 0 | Some concerns | Some concerns | No concerns | Some concerns | No concerns | No concerns | Low |
| NEd:RT | 0 | Some concerns | Some concerns | No concerns | Some concerns | Some concerns | No concerns | Very low |
| NEd:RT+SUP | 0 | Some concerns | Some concerns | No concerns | Major concerns | No concerns | No concerns | Low |
| NEd:SLEd | 0 | Some concerns | Some concerns | No concerns | Some concerns | Some concerns | No concerns | Very low |
| NEd:SUP | 0 | Some concerns | Some concerns | No concerns | No concerns | Some concerns | No concerns | Low |
| NEd+BT:PA | 0 | Some concerns | Some concerns | No concerns | Some concerns | No concerns | No concerns | Low |
| NEd+BT:RT | 0 | Major concerns | Some concerns | No concerns | Some concerns | No concerns | No concerns | Very low |
| NEd+BT:RT+SUP | 0 | Some concerns | Some concerns | No concerns | Major concerns | No concerns | No concerns | Very low |
| NEd+BT:SLEd | 0 | Major concerns | Some concerns | No concerns | Major concerns | No concerns | No concerns | Very low |
| NEd+BT:SUP | 0 | Major concerns | Some concerns | No concerns | Some concerns | No concerns | No concerns | Very low |
| PA:RT | 0 | Some concerns | Some concerns | No concerns | Some concerns | No concerns | No concerns | Low |
| PA:RT+SUP | 0 | Some concerns | Some concerns | No concerns | Major concerns | No concerns | No concerns | Very low |
| PA:SLEd | 0 | Some concerns | Some concerns | No concerns | Some concerns | No concerns | No concerns | Low |
| PA:SUP | 0 | Some concerns | Some concerns | No concerns | No concerns | Some concerns | No concerns | Low |
| RT:SLEd | 0 | Some concerns | Some concerns | No concerns | Major concerns | No concerns | No concerns | Very low |
| RT:SUP | 0 | Some concerns | Some concerns | No concerns | No concerns | Major concerns | No concerns | Very low |
| RT+SUP:SLEd | 0 | Some concerns | Some concerns | No concerns | Major concerns | No concerns | No concerns | Very low |
| RT+SUP:SUP | 0 | Some concerns | Some concerns | No concerns | Major concerns | No concerns | No concerns | Very low |
| SLEd:SUP | 0 | Some concerns | Some concerns | No concerns | Some concerns | No concerns | No concerns | Low |

*Note:* **AER**, Aerobic; **BT**, Behavioural Therapy; **CC**, Carbohydrate Counting; **CON**, Control; **DEd**, Diabetes Education; **FT**, Family Therapy; ***k***, number of studies; **MC**, Multicomponent; **NEd,** Nutritional Education; **PA**, Physical Activity; **RT**, Resistance Training; **SLEd,** Sleep Education; **SUP,** Nutritional Supplements.

**EMS Figure 1.** Risk of bias results (RoB 2).

**ESM Figure 2.** Network plot of available comparisons between different non-pharmacological interventions on insulin doses in children and adolescents with type 1 diabetes mellitus. The size of the nodes is directly proportional to the number of participants randomly assigned to each intervention. The width of the connecting lines corresponds to the number of studies comparing the respective interventions.


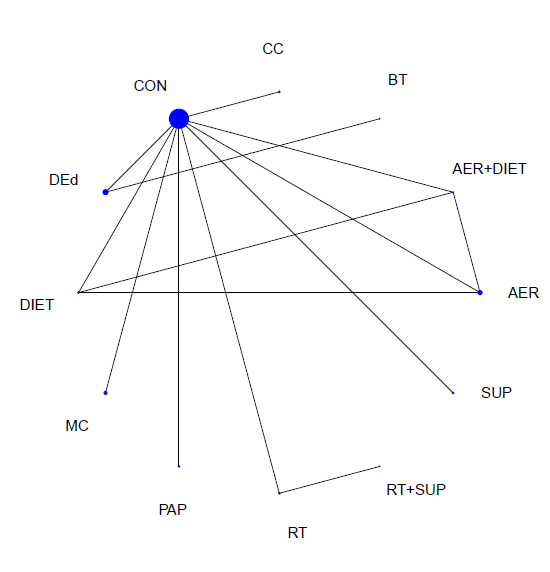


*Note:* **AER**, Aerobic; **BT**, Behavioural Therapy; **CC**, Carbohydrate Counting; **CON**, Control; **DEd**, Diabetes Education; **MC**, Multicomponent; **PA**, Physical Activity; **RT**, Resistance Training; **SUP,** Nutritional Supplements.

**ESM Figure 3.** Forest plot showing results on % glycated haemoglobin (HbA1c) after various non-pharmacological interventions compared to the control group, which received standard care. Each row represents a specific intervention, while the dots in the figure represent the change in % HbA1c, and the horizontal lines indicate 95% credible intervals. The model was estimated using a random effects model.


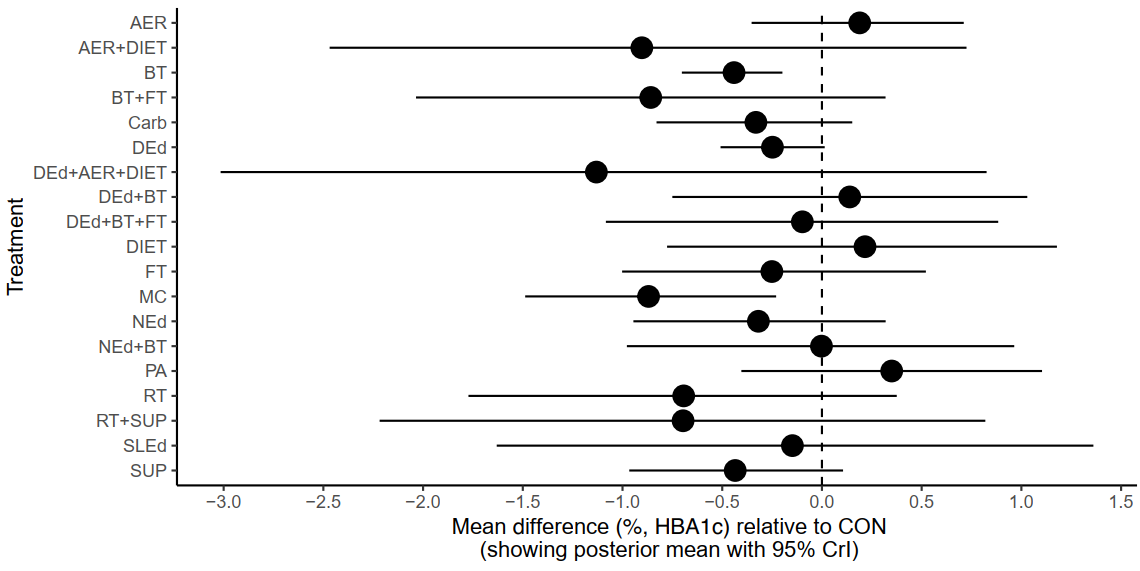


*Note:* **AER**, Aerobic; **BT**, Behavioural Therapy; **Carb**, Carbohydrate Counting; **CON**, Control; **CrI**, credible interval; **DEd**, Diabetes Education; **FT**, Family Therapy; **MC**, Multicomponent; **NEd,** Nutritional Education; **PA**, Physical Activity; **RT**, Resistance Training; **SLEd,** Sleep Education; **SUP,** Nutritional Supplements.

**ESM Figure 4.** Forest plot displaying results on daily insulin doses requirement for various non-pharmacological interventions compared to the control group, which received standard care. Each row represents a specific intervention, while the figure points represent the corresponding standardised mean, and the horizontal lines indicate 95% credible intervals. The model was calculated using a random-effects model.


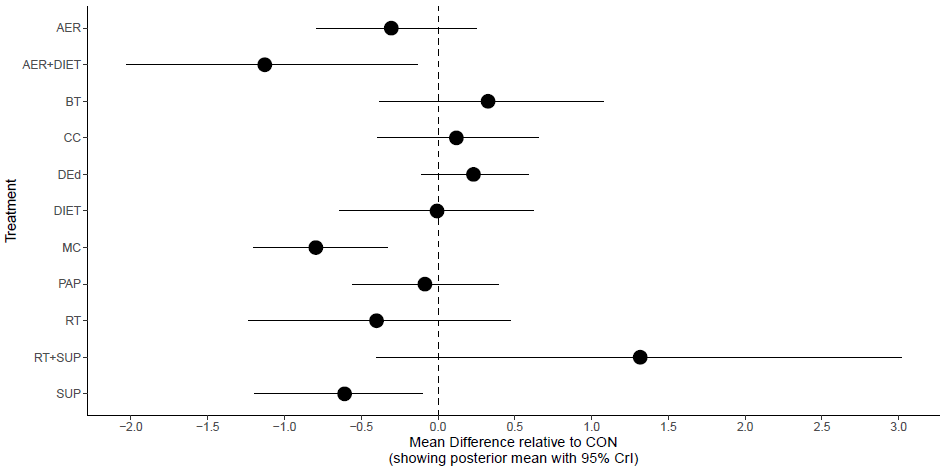


*Note:* **AER**, Aerobic; **BT**, Behavioural Therapy; **CC**, Carbohydrate Counting; **CON**, Control; **CrI**, credible interval; **DEd**, Diabetes Education; **MC**, Multicomponent; **PA**, Physical Activity; **RT**, Resistance Training; **SUP,** Nutritional Supplements.

**ESM Figure 5.** Rankogram for daily insulin doses requirement.


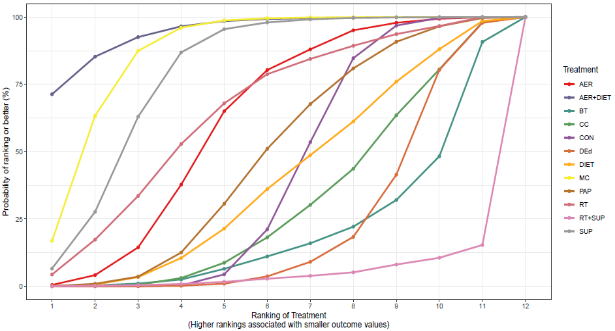


| **Rank** | AER | AER+DIET | BT | CC | CON | DEd | DIET | MC | PA | RT | RT + SUP | SUP |
| --- | --- | --- | --- | --- | --- | --- | --- | --- | --- | --- | --- | --- |
| 1 | 0.24 | 69.08 | 0.15 | 0.02 | 0 | 0 | 0.06 | 17.04 | 0.15 | 5.07 | 0.06 | 8.13 |
| 2 | 3 | 83.58 | 0.54 | 0.19 | 0 | 0 | 0.85 | 62.2 | 0.73 | 18.08 | 0.18 | 30.64 |
| 3 | 13.22 | 92.71 | 1.43 | 0.76 | 0.04 | 0.08 | 3.67 | 87.21 | 3.38 | 33.52 | 0.42 | 63.55 |
| 4 | 38.68 | 96.89 | 3.32 | 2.76 | 0.47 | 0.31 | 11.66 | 96.15 | 11.95 | 52.82 | 0.88 | 84.09 |
| 5 | 67.61 | 98.51 | 6.94 | 8.38 | 4.06 | 1.02 | 22.63 | 98.63 | 29.72 | 66.24 | 1.76 | 94.48 |
| 6 | 84.22 | 99.12 | 11.84 | 17.74 | 20.83 | 3 | 37.47 | 99.5 | 49.73 | 75.82 | 2.87 | 97.83 |
| 7 | 91.82 | 99.46 | 17.39 | 30.53 | 50.73 | 7.58 | 49.35 | 99.76 | 67.25 | 83.04 | 3.96 | 99.1 |
| 8 | 95.97 | 99.69 | 25.1 | 45.65 | 81.75 | 17.25 | 61.57 | 99.89 | 80.13 | 88.43 | 4.98 | 99.58 |
| 9 | 98.36 | 99.84 | 36.31 | 64.32 | 96.4 | 40.28 | 74.9 | 99.94 | 90.14 | 93.31 | 6.37 | 99.82 |
| 10 | 99.42 | 99.92 | 53.97 | 82.04 | 99.48 | 77.05 | 86.42 | 99.97 | 96.38 | 96.73 | 8.68 | 99.95 |
| 11 | 99.95 | 99.97 | 92.48 | 97.89 | 100 | 98.28 | 98.12 | 99.99 | 99.54 | 99.98 | 13.82 | 99.99 |
| 12 | 100.02 | 99.99 | 100 | 99.99 | 100 | 100.01 | 100.01 | 99.99 | 100 | 100 | 100 | 100 |
|  |  |  |  |  |  |  |  |  |  |  |  |  |
| **SUCRA** | 62.95 | 94.43 | 22.68 | 31.84 | 41.25 | 22.26 | 40.61 | 87.3 | 48.1 | 64.82 | 4 | 79.74 |

*Note:* **AER**, Aerobic; **BT**, Behavioural Therapy; **CC**, Carbohydrate Counting; **CON**, Control; **DEd**, Diabetes Education; **MC**, Multicomponent; **PA**, Physical Activity; **RT**, Resistance Training; **SUCRA**, Surface Under Cumulative Ranking; **SUP,** Nutritional Supplements.

**ESM Figure 6.** Bayesian funnel-plots for publication bias for all nonpharmacological interventions and those comparisons with more than 10 studies. The X-axis shows the reported standardised mean difference effect of studies comparing nonpharmacological interventions versus control (A), Behavioural therapy versus control (B), and Diabetes education versus control (C); while the Y-axis shows standard error. Points near the upper area show more precise estimates, while absence of skewness indicates a lower likelihood of reporting bias

**(A) (B) (C)**


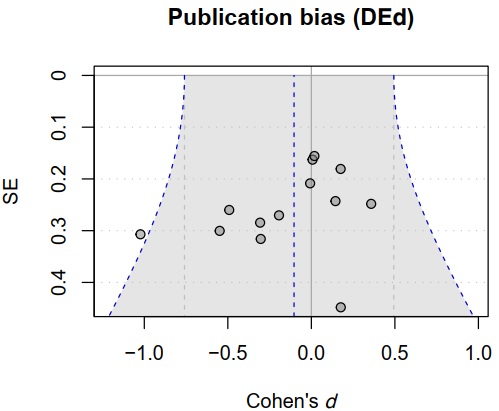

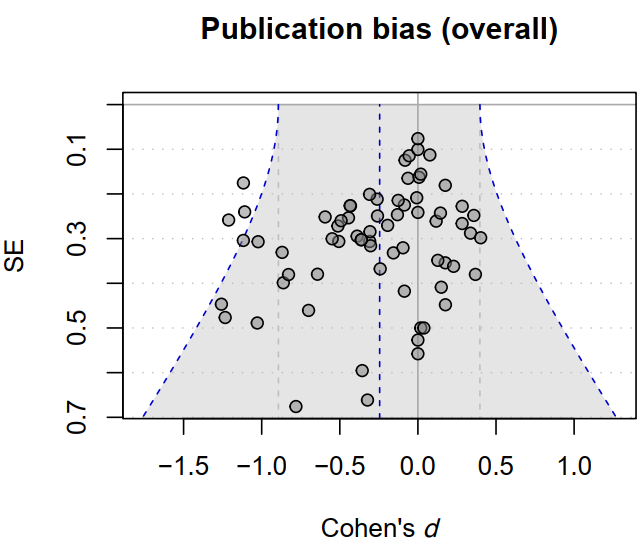

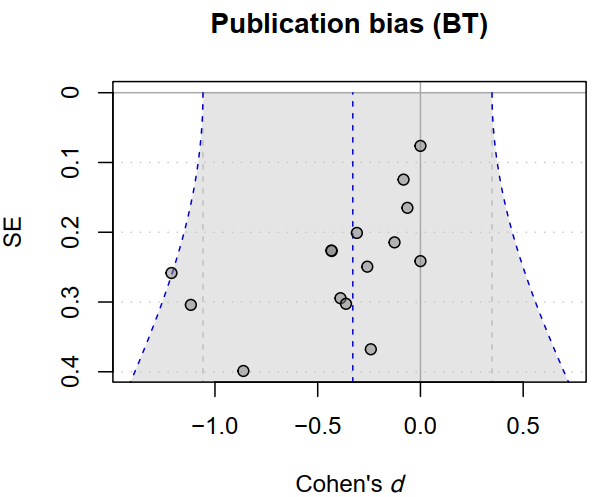


**ESM References**

1. Campaigne BN, Gilliam TB, Spencer ML, Lampman RM, Schork MA. Effects of a physical activity program on metabolic control and cardiovascular fitness in children with insulin-dependent diabetes mellitus. *Diabetes Care*. 1984;7(1):57-62. doi:10.2337/diacare.7.1.57

2. Campaigne BN, Landt KW, Mellies MJ, James FW, Glueck CJ, Sperling MA. The Effects of Physical Training on Blood Lipid Profiles in Adolescents With Insulin-Dependent Diabetes Mellitus. *Phys Sportsmed*. 1985;13(12):83-89. doi:10.1080/00913847.1985.11708949

3. D’hooge R, Hellinckx T, Van Laethem C, et al. Influence of combined aerobic and resistance training on metabolic control, cardiovascular fitness and quality of life in adolescents with type 1 diabetes: a randomized controlled trial. *Clin Rehabil*. 2011;25(4):349-359. doi:10.1177/0269215510386254

4. Gusso S, Pinto T, Baldi JC, et al. Exercise Training Improves but Does Not Normalize Left Ventricular Systolic and Diastolic Function in Adolescents With Type 1 Diabetes. *Diabetes Care*. 2017;40(9):1264-1272. doi:10.2337/dc16-2347

5. Hasan R, Perez-Santiago D, Churilla JR, et al. Can Short Bouts of Exercise (“Exercise Snacks”) Improve Body Composition in Adolescents with Type 1 Diabetes? A Feasibility Study. *Horm Res Paediatr*. 2019;92(4):245-253. doi:10.1159/000505328

6. Huttunen NP, Länkelä SL, Knip M, et al. Effect of once-a-week training program on physical fitness and metabolic control in children with IDDM. *Diabetes Care*. 1989;12(10):737-740. doi:10.2337/diacare.12.10.737

7. Knox E, Glazebrook C, Randell T, et al. SKIP (Supporting Kids with diabetes In Physical activity): Feasibility of a randomised controlled trial of a digital intervention for 9-12 year olds with type 1 diabetes mellitus. *BMC Public Health*. 2019;19(1):371. doi:10.1186/s12889-019-6697-1

8. Landt KW, Campaigne BN, James FW, Sperling MA. Effects of exercise training on insulin sensitivity in adolescents with type I diabetes. *Diabetes Care*. 1985;8(5):461-465. doi:10.2337/diacare.8.5.461

9. Mohammed MHH, Al-Qahtani MHH, Takken T. Effects of 12 weeks of recreational football (soccer) with caloric control on glycemia and cardiovascular health of adolescent boys with type 1 diabetes. *Pediatr Diabetes*. 2021;22(4):625-637. doi:10.1111/pedi.13203

10. Nazari M, Shabani R, Dalili S. The effect of concurrent resistance-aerobic training on serum cortisol level, anxiety, and quality of life in pediatric type 1 diabetes. *J Pediatr Endocrinol Metab JPEM*. 2020;33(5):599-604. doi:10.1515/jpem-2019-0526

11. Newton KH, Wiltshire EJ, Elley CR. Pedometers and text messaging to increase physical activity: randomized controlled trial of adolescents with type 1 diabetes. *Diabetes Care*. 2009;32(5):813-815. doi:10.2337/dc08-1974

12. Petschnig R, Wagner T, Robubi A, Baron R. Effect of Strength Training on Glycemic Control and Adiponectin in Diabetic Children. *Med Sci Sports Exerc*. 2020;52(10):2172-2178. doi:10.1249/MSS.0000000000002356

13. Salem MA, AboElAsrar MA, Elbarbary NS, ElHilaly RA, Refaat YM. Is exercise a therapeutic tool for improvement of cardiovascular risk factors in adolescents with type 1 diabetes mellitus? A randomised controlled trial. *Diabetol Metab Syndr*. 2010;2(1):47. doi:10.1186/1758-5996-2-47

14. Stratton R, Wilson DP, Endres RK, Goldstein DE. Improved glycemic control after supervised 8-wk exercise program in insulin-dependent diabetic adolescents. *Diabetes Care*. 1987;10(5):589-593. doi:10.2337/diacare.10.5.589

15. Tomar R, Hamdan M, Al-Qahtani MH. Effect of low to moderate intensity walking and cycling on glycaemic and metabolic control in type 1 diabetes mellitus adolescent males: A randomized controlled trial. *Isokinet Exerc Sci*. 2014;22(3):237-243. doi:10.3233/IES-140544

16. Tunar M, Ozen S, Goksen D, Asar G, Bediz CS, Darcan S. The effects of Pilates on metabolic control and physical performance in adolescents with type 1 diabetes mellitus. *J Diabetes Complications*. 2012;26(4):348-351. doi:10.1016/j.jdiacomp.2012.04.006

17. Wong CH, Chiang YC, Wai JPM, et al. Effects of a home-based aerobic exercise programme in children with type 1 diabetes mellitus. *J Clin Nurs*. 2011;20(5-6):681-691. doi:10.1111/j.1365-2702.2010.03533.x

18. Abdulrhman MM, El-Hefnawy MH, Aly RH, et al. Metabolic effects of honey in type 1 diabetes mellitus: A randomized crossover pilot study. *J Med Food*. 2013;16(1):66-72. doi:10.1089/jmf.2012.0108

19. Agrawal RP, Jain S, Shah S, Chopra A, Agarwal V. Effect of camel milk on glycemic control and insulin requirement in patients with type 1 diabetes: 2-years randomized controlled trial. *Eur J Clin Nutr*. 2011;65(9):1048-1052. doi:10.1038/ejcn.2011.98

20. Alfonsi JE, Choi EEY, Arshad T, et al. Carbohydrate counting app using image recognition for youth with Type 1 diabetes: Pilot randomized control trial. *JMIR MHealth UHealth*. 2020;8(10). doi:10.2196/22074

21. Altschuler JA, Casella SJ, MacKenzie TA, Curtis KM. The effect of cinnamon on A1C among adolescents with type 1 diabetes. *DIABETES CARE*. 2007;30(4):813-816. doi:10.2337/dc06-1871

22. Brown S, Lieberman D, Gemeny B, Fan Y, Wilson D, Pasta D. Educational video game for juvenile diabetes: Results of a controlled trial. *Med Inform (Lond)*. 1997;22(1):77-89. doi:10.3109/14639239709089835

23. Chatzakis C, Floros D, Papagianni M, et al. The Beneficial Effect of the Mobile Application *Euglyca* in Children and Adolescents with Type 1 Diabetes Mellitus: A Randomized Controlled Trial. *Diabetes Technol Ther*. 2019;21(11):627-634. doi:10.1089/dia.2019.0170

24. Donzeau A, Bonnemaison E, Vautier V, et al. Effects of advanced carbohydrate counting on glucose control and quality of life in children with type 1 diabetes. *Pediatr Diabetes*. 2020;21(7):1240-1248. doi:10.1111/pedi.13076

25. Duffus SH, Slaughter JC, Cooley W, et al. A pragmatic low carbohydrate diet intervention changes neither carbohydrate consumption nor glycemia in adolescents and young adults with type 1 diabetes in a randomized trial. *Pediatr Diabetes*. 2022;23(7):1088-1100. doi:10.1111/pedi.13407

26. Enander R, Gundevall C, Strömgren A, Chaplin J, Hanas R. Carbohydrate counting with a bolus calculator improves post-prandial blood glucose levels in children and adolescents with type 1 diabetes using insulin pumps. *Pediatr Diabetes*. 2012;13(7):545-551. doi:10.1111/j.1399-5448.2012.00883.x

27. Gökşen D, Atik Altınok Y, Özen S, Demir G, Darcan Ş. Effects of Carbohydrate Counting Method on Metabolic Control in Children with Type 1 Diabetes Mellitus. *J Clin Res Pediatr Endocrinol*. Published online June 5, 2014:74-78. doi:10.4274/jcrpe.1191

28. Kumar S, Kumar R, Rohilla L, Jacob N, Yadav J, Sachdeva N. A high potency multi-strain probiotic improves glycemic control in children with new-onset type 1 diabetes mellitus: A randomized, double-blind, and placebo-controlled pilot study. *Pediatr Diabetes*. 2021;22(7):1014-1022. doi:10.1111/pedi.13244

29. Ludvigsson J, Samuelsson U, Johansson C, Stenhammar L. Treatment with antioxidants at onset of type 1 diabetes in children: A randomized, double-blind placebo-controlled study. *Diabetes Metab Res Rev*. 2001;17(2):131-136. doi:10.1002/dmrr.176

30. Spiegel G, Bortsov A, Bishop FK, et al. Randomized Nutrition Education Intervention to Improve Carbohydrate Counting in Adolescents with Type 1 Diabetes Study: Is More Intensive Education Needed? *J Acad Nutr Diet*. 2012;112(11):1736-1746. doi:10.1016/j.jand.2012.06.001

31. Wang CH, Yen HR, Lu WL, et al. Adjuvant Probiotics of Lactobacillus salivarius subsp. salicinius AP-32, L. johnsonii MH-68, and Bifidobacterium animalis subsp. lactis CP-9 Attenuate Glycemic Levels and Inflammatory Cytokines in Patients With Type 1 Diabetes Mellitus. *Front Endocrinol*. 2022;13. doi:10.3389/fendo.2022.754401

32. Al Ksir K, Wood DL, Hasni Y, Sahli J, Quinn M, Ghardallou M. Motivational interviewing to improve self-management in youth with type 1 diabetes: A randomized clinical trial. *J Pediatr Nurs*. 2022;66:e116-e121. doi:10.1016/j.pedn.2022.05.001

33. Ambrosino JM, Fennie K, Whittemore R, Jaser S, Dowd MF, Grey M. Short-term effects of coping skills training in school-age children with type 1 diabetes. *Pediatr DIABETES*. 2008;9(3):74-82. doi:10.1111/j.1399-5448.2007.00356.x

34. Bakır E, Çavuşoğlu H, Mengen E. Effects of the Information–Motivation–Behavioral Skills Model on Metabolic Control of Adolescents with Type 1 Diabetes in Turkey: Randomized Controlled Study. *J Pediatr Nurs*. 2021;58:e19-e27. doi:10.1016/j.pedn.2020.11.019

35. Berndt RD, Takenga C, Preik P, et al. Impact of information technology on the therapy of type-1 diabetes: A case study of children and adolescents in Germany. *J Pers Med*. 2014;4(2):200-217. doi:10.3390/jpm4020200

36. Bloomfield S, Calder JE, Chisholm V, et al. A Project in Diabetes Education for Children. *Diabet Med*. 1990;7(2):137-142. doi:10.1111/j.1464-5491.1990.tb01348.x

37. Brorsson AL, Leksell J, Franko MA, Olinder AL. A person-centered education for adolescents with type 1 diabetes-A randomized controlled trial. *Pediatr DIABETES*. 2019;20(7):986-996. doi:10.1111/pedi.12888

38. Castensøe-Seidenfaden P, Husted GR, Jensen AK, et al. Testing a smartphone app (Young with diabetes) to improve self-management of diabetes over 12 months: Randomized controlled trial. *JMIR MHealth UHealth*. 2018;6(6). doi:10.2196/mhealth.9487

39. Channon SJ, Huws-Thomas MV, Rollnick S, et al. A Multicenter Randomized Controlled Trial of Motivational Interviewing in Teenagers With Diabetes. *Diabetes Care*. 2007;30(6):1390-1395. doi:10.2337/dc06-2260

40. Christie D, Thompson R, Sawtell M, et al. Effectiveness of a structured educational intervention using psychological delivery methods in children and adolescents with poorly controlled type 1 diabetes: A cluster-randomized controlled trial of the CASCADE intervention. *BMJ Open Diabetes Res Care*. 2016;4(1):1-14. doi:10.1136/bmjdrc-2015-000165

41. Edraki M, Zarei A, Soltanian M, Moravej H. The effect of peer education on self-care behaviors and the mean of glycosylated haemoglobin in adolescents with type 1 diabetes: A randomized controlled clinical trial. *Int J Community Based Nurs Midwifery*. 2020;8(3):209-219. doi:10.30476/ijcbnm.2020.82296.1051

42. Graue M, Wentzel-Larsen T, Hanestad BR, Sovik O. Evaluation of a programme of group visits and computer-assisted consultations in the treatment of adolescents with Type 1 diabetes. *Diabet Med*. 2005;22(11):1522-1529. doi:10.1111/j.1464-5491.2005.01689.x

43. Grey M, Yu C, Boland E, Sullivan-Bolyai S, Davidson M, Tamborlane W. Short-term effects of coping skills training as adjunct to intensive therapy in adolescents. *DIABETES CARE*. 1998;21(6):902-908. doi:10.2337/diacare.21.6.902

44. Grey M, Whittemore R, Jeon S, et al. Internet Psycho-Education Programs Improve Outcomes in Youth With Type 1 Diabetes. *DIABETES CARE*. 2013;36(9):2475-2482. doi:10.2337/dc12-2199

45. Han Y, Faulkner MS, Fritz H, et al. A Pilot Randomized Trial of Text-Messaging for Symptom Awareness and Diabetes Knowledge in Adolescents With Type 1 Diabetes. *J Pediatr Nurs*. 2015;30(6):850-861. doi:10.1016/j.pedn.2015.02.002

46. Hood KK, Iturralde E, Rausch J, Weissberg-Benchell J. Preventing Diabetes Distress in Adolescents With Type 1 Diabetes: Results 1 Year After Participation in the STePS Program. *Diabetes Care*. 2018;41(8):1623-1630. doi:10.2337/dc17-2556

47. Husted GR, Thorsteinsson B, Esbensen BA, et al. Effect of guided self-determination youth intervention integrated into outpatient visits versus treatment as usual on glycemic control and life skills: a randomized clinical trial in adolescents with type 1 diabetes. *Trials*. 2014;15(1):321. doi:10.1186/1745-6215-15-321

48. Ibrahim N, Treluyer JM, Briand N, Godot C, Polak M, Beltrand J. Text message reminders for adolescents with poorly controlled type 1 diabetes: A randomized controlled trial. *PLOS ONE*. 2021;16(3). doi:10.1371/journal.pone.0248549

49. Izquierdo R, Morin PC, Bratt K, et al. School-Centered Telemedicine for Children with Type 1 Diabetes Mellitus. *J Pediatr*. 2009;155(3):374-379. doi:10.1016/j.jpeds.2009.03.014

50. Jaser SS, Whittemore R, Choi L, Nwosu S, Russell WE. Randomized Trial of a Positive Psychology Intervention for Adolescents With Type 1 Diabetes. *J Pediatr Psychol*. 2019;44(5):620-629. doi:10.1093/jpepsy/jsz006

51. Jaser SS, Hamburger ER, Bergner EM, et al. Sleep coach intervention for teens with type 1 diabetes: Randomized pilot study. *Pediatr Diabetes*. 2020;21(3):473-478. doi:10.1111/pedi.12991

52. Katz ML, Volkening LK, Butler DA, Anderson BJ, Laffel LM. Family-based psychoeducation and care ambassador intervention to improve glycemic control in youth with type 1 diabetes: a randomized trial: Psychoeducation and care ambassador trial. *Pediatr Diabetes*. 2014;15(2):142-150. doi:10.1111/pedi.12065

53. Kaushal T, Katz LEL, Joseph J, et al. A Text Messaging Intervention With Financial Incentive for Adolescents With Type 1 Diabetes. *J Diabetes Sci Technol*. 2022;16(1):120-127. doi:10.1177/1932296820952786

54. Klee P, Bussien C, Castellsague M, et al. An Intervention by a Patient-Designed Do-It-Yourself Mobile Device App Reduces HbA1c in Children and Adolescents with Type 1 Diabetes: A Randomized Double-Crossover Study. *DIABETES Technol Ther*. 2018;20(12):797-805. doi:10.1089/dia.2018.0255

55. Lawson M, Cohen N, Richardson C, Orrbine E, Pham B. A randomized trial of regular standardized telephone contact by a diabetes nurse educator in adolescents with poor diabetes control. *Pediatr DIABETES*. 2005;6(1):32-40. doi:10.1111/j.1399-543X.2005.00091.x

56. Maranda L, Lau M, Stewart SM, Gupta OT. A Novel Behavioral Intervention in Adolescents With Type 1 Diabetes Mellitus Improves Glycemic Control: Preliminary Results From a Pilot Randomized Control Trial. *Diabetes Educ*. 2015;41(2):224-230. doi:10.1177/0145721714567235

57. Massouh SR, Steele TM, Alseth ER, Diekmann JM. The effect of social learning intervention on metabolic control of insulin-dependent diabetes mellitus in adolescents. *Diabetes Educ*. 1989;15(6):518-521. doi:10.1177/014572178901500609

58. Mayer-Davis EJ, Maahs DM, Seid M, et al. Efficacy of the Flexible Lifestyles Empowering Change intervention on metabolic and psychosocial outcomes in adolescents with type 1 diabetes (FLEX): a randomised controlled trial. *Lancet Child Adolesc Health*. 2018;2(9):635-646. doi:10.1016/S2352-4642(18)30208-6

59. Murphy HR, Wadham C, Hassler-Hurst J, Rayman G, Skinner TC, on behalf of Families and Adolescents Communication and Teamwork Study (FACTS) Group. Randomized trial of a diabetes self-management education and family teamwork intervention in adolescents with Type 1 diabetes: Families and Adolescents Communication and Teamwork Study 2 (FACTS 2). *Diabet Med*. 2012;29(8):e249-e254. doi:10.1111/j.1464-5491.2012.03683.x

60. Najmi SB, Marasi MR, Hashemipour M, Hovsepian S, Ghasemi M. The perceived self-efficacy and its interrelation with communication in family and glycemic control in adolescents with type 1 diabetes. *Pak J Med Sci*. 2013;29(1 SUPPL.):334-339. doi:10.12669/pjms.291(Suppl).3528

61. Nunn E, King B, Smart C, Anderson D. A randomized controlled trial of telephone calls to young patients with poorly controlled type 1 diabetes. *Pediatr Diabetes*. 2006;7(5):254-259. doi:10.1111/j.1399-5448.2006.00200.x

62. Panagiotopoulos C, Preston JM, Stewart LL, Metzger DL, Chanoine JP. Weekly Telephone Contact by a Diabetes Educator in Adolescents with Type 1 Diabetes. *Can J Diabetes*. 2003;27(4):422-427.

63. Price KJ, Knowles JA, Fox M, et al. Effectiveness of the Kids in Control of Food (KICk-OFF) structured education course for 11-16 year olds with Type 1 diabetes. *Diabet Med*. 2016;33(2):192-203. doi:10.1111/dme.12881

64. Robling M, McNamara R, Bennert K, et al. The effect of the Talking Diabetes consulting skills intervention on glycaemic control and quality of life in children with type 1 diabetes: cluster randomised controlled trial (DEPICTED study). *BMJ-Br Med J*. 2012;344. doi:10.1136/bmj.e2359

65. Saßmann H, De Hair M, Danne T, Lange K. Reducing stress and supporting positive relations in families of young children with type 1 diabetes: A randomized controlled study for evaluating the effects of the DELFIN parenting program. *BMC Pediatr*. 2012;12(1):152. doi:10.1186/1471-2431-12-152

66. Schache KR, Hofman PL, Serlachius AS. A pilot randomized controlled trial of a gratitude intervention for adolescents with Type 1 diabetes. *Diabet Med*. 2020;37(8):1352-1356. doi:10.1111/dme.14078

67. Schiaffini R, Tagliente I, Carducci C, et al. Impact of long-term use of eHealth systems in adolescents with type 1 diabetes treated with sensor-augmented pump therapy. *J Telemed Telecare*. 2016;22(5):277-281. doi:10.1177/1357633X15598425

68. Serlachius AS, Scratch SE, Northam EA, Frydenberg E, Lee KJ, Cameron FJ. A randomized controlled trial of cognitive behaviour therapy to improve glycaemic control and psychosocial wellbeing in adolescents with type 1 diabetes. *J Health Psychol*. 2016;21(6):1157-1169. doi:10.1177/1359105314547940

69. Stanger C, Lansing AH, Scherer E, Budney A, Christiano AS, Casella SJ. A Web-Delivered Multicomponent Intervention for Adolescents with Poorly Controlled Type 1 Diabetes: A Pilot Randomized Controlled Trial. *Ann Behav Med*. 2018;52(12):1010-1022. doi:10.1093/abm/kay005

70. Tuomaala AK, Hero M, Tuomisto MT, et al. Motivational Interviewing and Glycemic Control in Adolescents With Poorly Controlled Type 1 Diabetes: A Randomized Controlled Pilot Trial. *Front Endocrinol*. 2021;12:639507. doi:10.3389/fendo.2021.639507

71. Wang YC, Stewart SM, Mackenzie M, Nakonezny PA, Edwards D, White PC. A Randomized Controlled Trial Comparing Motivational Interviewing in Education to Structured Diabetes Education in Teens With Type 1 Diabetes. *DIABETES CARE*. 2010;33(8):1741-1743. doi:10.2337/dc10-0019

72. Whittemore R, Liberti LS, Jeon S, et al. Efficacy and implementation of an Internet psychoeducational program for teens with type 1 diabetes. *Pediatr DIABETES*. 2016;17(8):567-575. doi:10.1111/pedi.12338

73. Wysocki T, Harris MA, Buckloh LM, et al. Effects of behavioral family systems therapy for diabetes on adolescents’ family relationships, treatment adherence, and metabolic control. *J Pediatr Psychol*. 2006;31(9):928-938. doi:10.1093/jpepsy/jsj098

74. Zarifsaniey N, Shirazi MO, Mehrabi M, Bagheri Z. Promoting self-management behaviors in adolescents with type 1 diabetes, using digital storytelling: a pilot randomized controlled trial. *BMC Endocr Disord*. 2022;22(1):74. doi:10.1186/s12902-022-00988-7

75. Katz ML, Volkening LK, Butler DA, Anderson BJ, Laffel LM. Family-based psychoeducation and care ambassador intervention to improve glycemic control in youth with type 1 diabetes: a randomized trial. *Pediatr DIABETES*. 2014;15(2):142-150. doi:10.1111/pedi.12065

76. Whittemore R, Liberti LS, Jeon S, et al. Efficacy and implementation of an Internet psychoeducational program for teens with type 1 diabetes: Internet psychoeducation for teens with T1D. *Pediatr Diabetes*. 2016;17(8):567-575. doi:10.1111/pedi.12338

77. Tomar R, Hamdan M, Al-Qahtani MH. Effect of low to moderate intensity walking and cycling on glycaemic and metabolic control in type 1 diabetes mellitus adolescent males: A randomized controlled trial. *Isokinet Exerc Sci*. 2014;22(3):237-243.

78. Channon SJ, Huws-Thomas MV, Rollnick S, et al. A multicenter randomized controlled trial of motivational interviewing in teenagers with diabetes. *DIABETES CARE*. 2007;30(6):1390-1395. doi:10.2337/dc06-2260

79. Izquierdo M. Prescripción de ejercicio físico. El programa Vivifrail como modelo. *Nutr Hosp*. Published online 2019. doi:10.20960/nh.02680

80. Agrawal RP, Jain S, Shah S, Chopra A, Agarwal V. Effect of camel milk on glycemic control and insulin requirement in patients with type 1 diabetes: 2-years randomized controlled trial. *Eur J Clin Nutr*. 2011;65(9):1048-1052. doi:10.1038/ejcn.2011.98
